# Metabolic Functions and Mechanisms of Selenium, Selenocysteine, and GPX4 Mediated Immune Regulation Through Autophagy in Solid Tumors

**DOI:** 10.1002/fsn3.71230

**Published:** 2025-11-27

**Authors:** Liang Yang, Yu Jiang, Haoyue Wu, Jinnan Sun, Xinyue Huang, Jingfeng Hong, Bin Zheng, Jinmiao Zhu

**Affiliations:** ^1^ School of Chemistry and Pharmaceutical Engineering Hefei Normal University Hefei China; ^2^ Anhui Zhongzhi Technology Co., Ltd Hefei China

**Keywords:** autophagy, GPX4, immunity, selenium, selenium metabolism, solid tumors

## Abstract

Selenium, an essential trace element in the human body, is present in the form of selenocysteine (Sec) within 25 selenoproteins, including selenoprotein N, selenoprotein P, and glutathione peroxidases (GPXs). An increasing number of studies have shown that selenoproteins resulting from selenium metabolism exert a significant effect on diverse immune cells. For instance, both selenoprotein K and GPX4 are intricately involved in the initiation and resolution of pro‐inflammatory responses in granulocytes (PMN). The function of dendritic cells (DCs) exhibits a specific association with the expression of selenoproteins. Methionine sulfoxide reductase B1 (MSRB1) can facilitate lipopolysaccharide (LPS) in stimulating bone marrow‐derived macrophages (BMDM), induce the production of anti‐inflammatory cytokines interleukin‐10 (IL‐10) and interleukin‐1 receptor antagonist (IL‐1RA), and thereby partake in immune activities. The roles of selenoproteins in immune cells underscore their significance and complexity in the overall physiological process. In particular, their involvement in tumor immunity warrants in‐depth exploration. In solid tumors, the process by which cancer cells generate selenoproteins, especially GPX4, through selenium metabolism constructs a defense mechanism against ferroptosis. This process is highly reliant on the capacity of cancer cells to take up selenium independently, as well as the activation of selenium metabolic pathways such as the trans‐selenation pathway and the breakdown of inorganic selenium compounds. In the autophagy mediated by copper and erastin (a ferroptosis inducer), the autophagy receptors TAX1BP1 and SQSTM1 can promote the degradation of GPX4, effectively reducing the resistance of cancer cells to ferroptosis. This distinctive mechanism has opened up a novel perspective for research and offered potential therapeutic targets in the field of cancer treatment. In this review, we conduct a comprehensive and in‐depth analysis of the roles played by selenoproteins derived from selenium metabolism in the regulation of immune cells associated with immune diseases. Moreover, we elaborate in detail on the effects of GPX4 in relation to ferroptosis in solid tumors under the influence of autophagy‐mediated immunomodulation.

## Graphical abstract

This paper focuses on selenium, selenocysteine, and GPX4, elaborating on their metabolic functions and mechanisms in mediating immune regulation through autophagy in solid tumors. It explores the characteristics of selenium metabolism in tumors and the roles of related enzymes, providing new directions for cancer treatment. The article reviews the roles of selenium and related selenoproteins in immune regulation, analyzes their metabolic processes in solid tumors, and particularly emphasizes the association between GPX4, ferroptosis, and autophagy, offering a theoretical basis for tumor research and treatment. This paper comprehensively analyzes the regulatory effects of selenoproteins produced by selenium metabolism on immune cells, elaborates in detail on the crucial impact of GPX4 on ferroptosis in solid tumors and the regulatory mechanism of autophagy on it, providing new ideas for cancer treatment strategies.

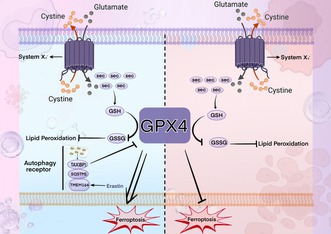

## 1 Introduction

As a dietary trace mineral, selenium (Se) exerts multiple physiological functions in humans including antioxidant, immunomodulatory, and neuroprotective effects (Rayman 2012), and is also widely utilized in the food industry as a nutritional fortificant for the development of functional foods. For example, selenium derivatives such as selenocysteine (Sec) and Se‐methylselenocysteine (MSC) have been authorized for use in various foods and dietary supplements to meet human selenium requirements, particularly in selenium‐deficient regions where they contribute to improved health outcomes. Regulatory frameworks in China, the United States, and the European Union—namely China's National Food Safety Standard (GB 1903.12‐2015), the U.S. Food and Drug Administration (FDA; Food Safety Modernization Act, FSMA), and the European Food Safety Authority (EFSA) provide explicit provisions governing the use of these selenium derivatives to ensure safety while maximizing their health benefits. Humans primarily obtain organic selenium from foods such as fish, shellfish, and animal offal, with Brazil nuts being an exceptionally rich source (Hadrup and Ravn‐Haren 2023). Additionally, dietary supplements commonly contain inorganic selenium (See et al. 2006). The dietary supply and bioavailability of selenium in humans depend largely on its organic forms. As an essential trace element, the protective role of selenium in various physiological processes has become a major research focus. Experts note that selenium plays a critical role in maintaining male fertility and delaying aging, among other functions. Acting as an endogenous antioxidant, it can regulate the endocrine system by modulating the dynamic balance of enzymes and metabolites such as glutathione peroxidase (GPX), superoxide dismutase (SOD), malondialdehyde (MDA), and catalase (CAT) (Barchielli et al. 2022; Kieliszek et al. 2022). Both selenium deficiency and excess are detrimental to human health. Selenium deficiency is associated with the development of chronic diseases (Avery and Hoffmann 2018), including certain human cancers such as breast, lung, gastric, bladder, ovarian, pancreatic, thyroid, esophageal, head and neck, cerebellar cancers, and melanoma (Kim, Choi, et al. 2021).

To explore the potential therapeutic value of selenoproteins in tumor immunity, this review is based on the core hypothesis that “the mechanisms of ferroptosis resistance in cancer cells mediated by selenium and selenoproteins can be therapeutically targeted.” We focus on the process by which cancer cells in solid tumors synthesize key selenoproteins (such as GPX4) through selenium metabolism to establish a defense system against ferroptosis. In recent years, an increasing number of metabolic pathways have been found to be associated with ferroptosis. Biochemically, ferroptosis is characterized by the generation of lethal levels of iron‐dependent lipid peroxidation (Xie et al. 2016; Stockwell et al. 2017). It has attracted considerable attention due to its close links with diseases such as ischemia–reperfusion injury (IRI), neurological disorders, and cancer (Stockwell et al. 2017; Li et al. 2019; Chen, Kang, et al. 2021; Ma et al. 2022). Iron is an essential nutrient for cell proliferation and a cofactor for many metabolic enzymes; thus, elevated iron levels can promote tumorigenesis and growth. Moreover, ferroptosis may trigger tumor initiation in the early stages by increasing inflammatory responses (Dai et al. 2020). Therefore, inducing ferroptosis to deplete iron could serve as an effective strategy to eliminate various cancer cells in advanced stages (Hassannia et al. 2019). In numerous studies targeting ferroptosis and autophagy in cancer cells, GPX4 has emerged as a key factor, with its activity playing a crucial role in solid tumors and related diseases (Xie et al. 2023; Zhang et al. 2024). However, current research has not yet explored the connection between autophagy and ferroptosis mediated specifically via GPX4.

This review investigates the roles of metabolic pathways—including selenium uptake in cancer cells, activation of the trans‐selenation pathway, and decomposition of inorganic selenium compounds—in this defense mechanism. Furthermore, it examines the molecular mechanism by which the autophagy receptors TAX1BP1 and SQSTM1 induce GPX4 degradation and impair cancer cell resistance to ferroptosis during copper‐ and erastin‐mediated autophagy processes. The aim is to identify novel therapeutic targets and strategies for cancer treatment.

## 2 Methodology

### 2.1 Search Strategy

Specific literature and title/abstract searches were conducted in PubMed, Scopus, Web of Science, and Science Direct databases to determine selenium enrichment. Search terms included “selenium”, “immunity,” “autophagy,” “selenocysteine,” “solid tumor,” and “GPX4.” Filters only identified studies published in the last decade. Non‐duplicate records were identified and filtered for title and abstract.

### 2.2 Search and Article Redirection

In the first section of this article, it was decided to focus on selenium to explore its protein types and functions and the effect of selenium enrichment on the immune mechanism. The terms “selenium,” “immunity,” and “selenoproteins” were used to search the literature. Keywords: “selenium,” “immunity,” and “selenoprotein.” The main research direction of the article is selenium metabolism and the role of proteins produced by selenium metabolism in the immune mechanism.

The second section of this article, focuses on the role of selenium metabolism and the selenoprotein GPX4 produced by selenium metabolism in tumors, and the keywords of the search were added to “tumor” and “GPX4”.

In the third part of this review, the word “tumor” in the second section was changed to “solid tumor,” and the keyword “autophagy” was added. The research direction is selenium metabolism and the role of GPX4 in immunomodulation mediated by autophagy. We will also focus on the role of selenium metabolism and GPX4 in solid tumors through autophagy‐mediated immunomodulation.

**FIGURE 1:**
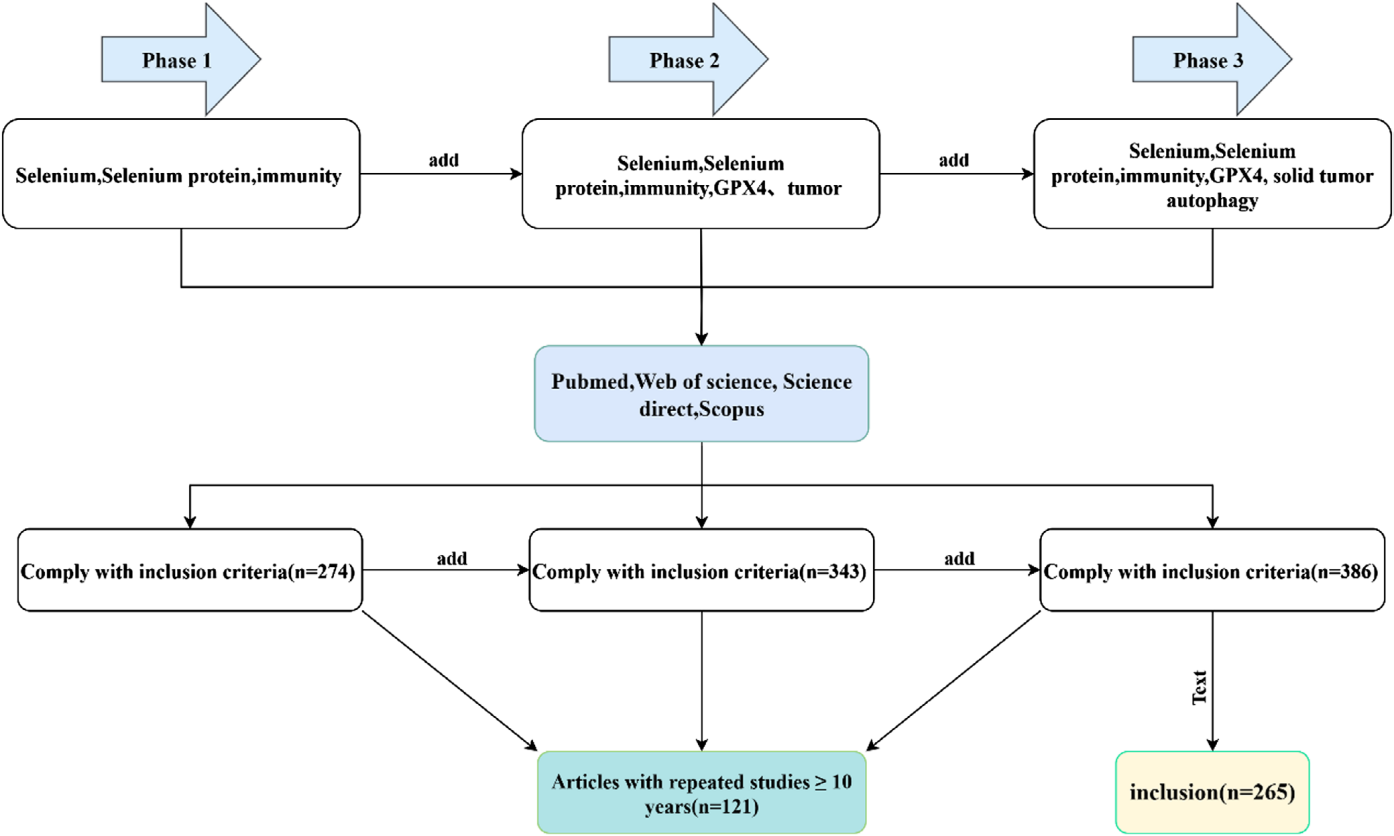
Study flowchart.

A summary of the relevant data from the included papers is shown in Figure 1.

### 2.3 Results

During the process of data screening and literature refinement, articles relevant to the target keywords were identified. Following the removal of duplicates and studies unrelated to the research focus, 265 articles were ultimately identified as eligible for analysis. Figure 1 depict the study screening process.

## 3 Trace Element Selenium

Selenium (Se), an essential trace element present in diverse compounds, was discovered in 1817 by Jöns Jakob Berzelius and named after Selene, the Greek moon goddess. In 1957, Schwartz and Folz first demonstrated the protective effect of selenium on organisms (Kieliszek and Błażejak 2016). Through extensive research, it has been established that selenium exerts profound effects on multiple human systems and physiological functions. These encompass the maintenance of muscle function, the normal operation of male reproductive biology, the stability of the cardiovascular system, the balanced regulation of the endocrine system, and the healthy development of the nervous system. Particularly within the immune system, selenium plays a critical role. The mechanisms underlying its actions in immune modulation processes, such as anti‐inflammation and antioxidation, have consistently been a major focus of scientific investigation.

The biological activity of selenium is intrinsically linked to its chemical forms in nature. Research indicates that selenium primarily exists in two distinct states within biological systems: organic and inorganic. Selenocysteine (SeCys) and selenomethionine (SeMet) are recognized as the principal organic forms of selenium. Conversely, elemental selenium, selenite, selenate, and selenides constitute the inorganic forms. Humans meet their physiological selenium requirements through the dietary intake of these two classes of selenium compounds. It is noteworthy that selenium possesses a narrow therapeutic range. It has been discovered that both selenium deficiency and excess exert adverse effects on human health. The association between selenium status and disease risk demonstrates a characteristic U‐shaped curve relationship (Rayman 2020). We observed that under conditions of selenium insufficiency, the hierarchical regulatory mechanisms governing selenium distribution within organisms can mitigate the effects of deficiency by prioritizing critical tissues and biochemical pathways (Schomburg 2021). This enables individuals in low‐selenium states to often exhibit no overt phenotypic alterations or discernible health symptoms. Without laboratory testing, this latent deficient state is easily overlooked. However, large‐scale analytical studies indicate that when selenium intake falls below recommended levels, disease risk significantly increases, particularly among individuals with chronic diseases, inflammatory responses, or other susceptibility factors (Rayman 2008; Schomburg 2020). Selenium deficiency has been recognized as a contributing factor to various pathophysiological conditions, including Keshan disease (Xu et al. 1997; Chen 2012), neuromuscular disorders (Moghadaszadeh et al. 2001), cancer (Yakubov et al. 2014), and male infertility (Mistry et al. 2012). Furthermore, studies suggest that low systemic selenium levels are associated with an elevated risk of neurodegenerative diseases (such as Alzheimer's disease and Parkinson's disease) and autoimmune disorders (such as Hashimoto's thyroiditis and Graves' disease) (Li et al. 2025; Knezevic et al. 2020).

Conversely, selenium excess promotes oxidative stress and DNA damage, potentially increasing carcinogenic risk. Selenium also significantly influences hormone metabolism, particularly thyroid function; its presence is essential for the activity of deiodinases, which convert thyroxine (T4) to triiodothyronine (T3) (Knezevic et al. 2020; Arthur et al. 1993). Recent randomized trials suggest a positive correlation between excessive selenium intake and type 2 diabetes as well as advanced prostate cancer (Vinceti et al. 2018). Although selenium toxicity (selenosis) is considerably less common than deficiency, it can affect individuals due to over‐supplementation (MacFarquhar et al. 2010). Cases have been documented in regions such as Enshi (Hubei, China), Punjab (India), and South Dakota (USA), where populations exposed to exceptionally high dietary selenium levels exhibited characteristic manifestations including brittle hair; thickened, brittle, and lamellated nails; and in some cases, a garlic‐like odor on breath and skin (MacFarquhar et al. 2010; Rayman 2008). Other symptoms, such as vomiting and pulmonary edema, are characteristic of acute selenium poisoning (Fairweather‐Tait et al. 2011). Many instances of selenium excess in humans and livestock within specific geographical areas are typically influenced by high soil selenium concentrations. Animals grazing on fields with selenium concentrations exceeding 5 μg/g can accumulate levels harmful to humans. Selenium derivatives exhibit varying levels of toxicity, with inorganic forms possessing greater toxicity potential compared to organic forms (Dhanya et al. 2014). This implies that both insufficient and excessive selenium intake can disrupt the body's internal homeostasis, consequently elevating susceptibility to disease. Consequently, selenium supplementation necessitates precise control and rigorous monitoring.

According to data provided by the World Health Organization (WHO), the recommended daily selenium intake for adult women is 55 μg, while for adult men it is 70 μg. Concurrently, based on findings from various sources, the upper tolerable intake level for selenium ranges approximately between 300 and 600 μg/day (Stoffaneller and Morse 2015; Tutel'ian 2009). Furthermore, studies citing safety intake recommendations indicate that approximately 800 μg Se/day represents the no‐observed‐adverse‐effect level (NOAEL) (Stoffaneller and Morse 2015). Yang et al. evaluated the safety profile of L‐selenomethylcysteine (L‐SeMC) as a nutritional fortifier and recommended a safe intake of 3.4 mg/kg/day when used as a dietary supplement (Yang and Jia 2014). The selenium content in the soil of Middle Eastern countries (e.g., Saudi Arabia: 0.1–0.11 mg/kg; Libya: 0.09–0.62 mg/kg) is significantly lower than the global average (0.4 mg/kg), leading to inadequate dietary selenium intake (Kieliszek et al. 2022). These data strongly indicate that maintaining selenium intake within an appropriate range is crucial for safeguarding human health.

## 4 Selenoproteins

Selenoproteins represent a distinct class of proteins that contain one or more selenocysteine residues. Their synthesis mechanism is rather unique, involving a diverse array of specific enzymes and factors, and is intricately linked to selenium intake. Although selenoproteins are somewhat sporadically distributed within organisms, they are ubiquitously present across all life forms. Once selenium from food is ingested by humans, it predominantly exists in the body in the natural organic forms of selenocysteine and selenoproteins. By employing bioinformatics to analyze sequenced genomes and other DNA sources in an effort to explore the characteristics of genes encoding selenoproteins, at least 50 distinct selenoprotein families, comprising at least 837 individual selenoproteins, have been identified in nature (Arnér 2020). Humans and rodents possess 25 and 24 known selenoproteins, respectively. These proteins exhibit an extremely complex and diverse range of functions within organisms (Papp et al. 2007) (Table 1). For instance, when researchers investigated the differences in selenoprotein expression among various organisms, they discovered that GPX1, GPC4, SELENOF, SELENOK, SELENOM, SELENOS, and SELENOW were highly expressed in mice, while SELENOW and SELENOF had the highest expression levels in humans (Sasuclark et al. 2019). Accordingly, this review will provide a concise introduction to seven selenoproteins (SELENON, SELENOP, SELENOK, SELENOS, SELENOW, SELENOM, and GPX4) that exhibit relatively high expression levels in human tissues. Furthermore, it will present the expression profiles, molecular mechanisms, and clinical implications of the 25 selenoproteins across various cancers (Table 2).

**TABLE 1:** The distribution of some selenoproteins can be searched in the Human Protein Atlas (HPA) (website: https://www.proteinatlas.org/). As a comprehensive resource platform, HPA enables the exploration of the expression and distribution patterns of 16,975 individual proteins across all cells, tissues, and organs in the human body.

Selenoprotein	Presence of subcellular species	Genera present	Expression in tissues and organs	Function (reference)
SelenoP	Secreted proteins, plasma	Human, rat	Secreted by liver to plasma, also expressed in other tissue cells	Participate in oxidation Reduction Adjustment (Kryukov et al. 2003)
SelenoN	ER (endoplasmic reticulum) membrane	Human	It's found all over the body	Conditioning Regeneration (Moghadaszadeh et al. 2001)
SelenoK	Immune cells, ER membranes	Human, mice and chickens	Mainly expressed in heart and skeletal muscle, spleen	Antioxidant properties; Ca^2+^ flux regulation; Immunomodulation; Apoptosis regulation (Thu et al. 2010)
SelenoM	ER membranes and nerve cells	Human, mouse	Highest expression in the brain also found in the thyroid gland heart and other organs	Antioxidant activity (Nunes et al. 2023)
SelenoS	Mainly in the endoplasmic reticulum and plasma membrane, with small amounts in the Golgi apparatus	Human, mouse, pig	Spleen, blood vessels, serum	Modulation of inflammatory responses and removal of misfolded proteins from the endoplasmic reticulum induces stress apoptosis in the endoplasmic reticulum (Santos, Durães, et al. 2019; Zhu et al. 2022)
SelenoW	Mainly in the cytoplasm, a small portion in the cell membrane	Human, mouse	Skeletal muscle, colon Heart and prostate	Antioxidants for Human Lungs (Yao et al. 2013)
SelenoI	Plasma membrane	Human, mouse	Prevalent in all parts of the body voice (an opinion)	T‐cell activation of key CNS enzymes (Ma et al. 2021); Neurodevelopment (Nunes et al. 2022)
SelenoH	Selenoprotein H (SelenoH)	Human, mouse	Prevalent in all parts of the body voice (an opinion)	Gene regulation of glutathione (Mehta et al. 2013)
SelenoF	ER membrane	Human, mouse	Brain, prostate, testes, liver, and kidneys	Immunomodulation; regulates glycogenolysis and lipogenesis (Zhang et al. 2022)
SelenoT	Golgi and ER membranes	Human, mouse	cardiovascular	Regulation of oxidative stress; mitigation of ER stress; modulation of endoplasmic reticulum mitochondrial Ca^2+^ flux (Pothion et al. 2020)
SelenoV	ER membrane	Human, mouse	Testicles, liver	Protects against pro‐oxidant‐induced endoplasmic net stress and oxidative damage (Zhang et al. 2020)
SelenoO	Mitochondrial membrane	Human, mouse	cartilaginous tissue	Promotes chondrocyte viability, proliferation, and chondrogenic differentiation (Yan et al. 2016)
MSRB1	Cytoplasm, nucleus	Human, mouse	Prevalent, mainly in the liver	Antioxidant activity. Protein repair; regulation of immunity (Lee et al. 2017)
SEPHS2	Hepatocyte	Human, mouse	Kidney, liver	As a selenium donor for Sec (Nunziata et al. 2019) Regulates tumor growth (Zhang et al. 2021)
GPX1	Erythrocyte, cytoplasm, mitochondria, plasma membrane	Human, mouse	Found throughout the body, but higher in bones and liver	Reduction of intracellular H_2_O_2_ (Lubos et al. 2011; Lei et al. 2007)
GPX2	Cytoplasm, gastrointestinal tissue	Human, mouse	Mainly found in gastrointestinal tissues and human liver	Reduces peroxides in the gut (Brigelius‐Flohé and Kipp 2009)
GPX3	Plasma, exosomes	Human, mouse	Plasma, kidney, thyroid	Reduction of H_2_O_2_ in lipids, plasma, and thyroid cells (Gong et al. 2019; Hauffe et al. 2020)
GPX4	Cytoplasm, mitochondria, nucleus	Human, mouse, bat	Highly expressed in the testis	Antioxidant in brain membranes, promotes sperm motility, reduces complex lipid compounds (Pei et al. 2023), Regulates iron apoptosis (Xue et al. 2023)
GPX6	Secretory protein	Human	Embryonic tissues and epithelial cells of the olfactory organs	Inhibiting oxidative stress (Pei et al. 2023)
TXNRD1	Cytoplasm and nucleus	Human, mouse	ubiquitous	Antioxidant activity; Regenerating thioredoxin; Inhibiting cellular iron death (Hao et al. 2024; Cheff et al. 2023)
TXNRD2	Mitochondria	Human, mouse	Testis‐specific expression	Regeneration of thioredoxin; Regulation of cell proliferation and apoptosis (Yoshioka 2015)
TXNRD3	Mitochondria	Human, mouse	testicular	Antioxidant activity; inhibits apoptosis (Dou et al. 2022)
DIO1	Plasma membrane	Human, mouse	Liver, kidney, thyroid	Promotes production of active T3 cell hormones in the thyroid and peripheral tissues (Bingheng 2008)
DIO2	ER membrane	Human, mouse	Highly expressed in central nervous system, brown adipose tissue and skeletal muscle, pituitary gland, heart	Key regulators of human cardiomyocyte and mitochondrial performance (Bomer et al. 2021)
DIO3	Plasma membrane	Human, mouse	Present in placenta, uterus, fetus, skin, cerebral cortex, and CNS	Preventing high fetal exposure to CD4T3 cells. Thyroid Hormone Inactivation (Rasmussen et al. 2011)

**TABLE 2:** Mechanisms and clinical implications of 25 selenoproteins in various tumors.

Selenoprotein	Expression status	Associated cancer types	Molecular actions and mechanisms	Clinical significance/applications
SEPHS2	Upregulated (cancer‐specific)	BRCA, PCA	Catalyzes selenophosphate synthesis and detoxifies endogenous selenides (H_2_Se)	Knockdown inhibits tumor growth; specific targeting of cancer cells (Carlisle et al. 2020)
SelenoP	Upregulated (CRC), downregulated (HCC)	CRC, HCC	In CRC, SELENOP enhances the WNT/β‐catenin pathway by binding to WNT3A and LRP5/6, accelerating APC loss‐driven tumorigenesis; in HCC, low SELENOP expression is associated with lipid metabolism disorders (downregulation of PPARA/APOC3), abnormal hormone receptors, impaired antioxidant capacity, and promotion of hypoxic microenvironment	In CRC, SELENOP can serve as a potential therapeutic target, and intervention can be achieved by blocking the SELENOP‐LRP5/6 interaction via heparin; low expression in HCC is positively correlated with advanced staging and hypoxia scores (Prabhu 2023; Razaghi and Björnstedt 2024)
SelenoN	Upregulated	ESCC	Upregulation of SelN induces endoplasmic reticulum calcium overload by interfering with SERCA, activates the IRE1α (S724)‐CHOP‐BCL2 pathway to induce apoptosis, and synergistically enhances the tumor metastasis inhibitory effect of Nab‐PTX	Combination of LNT‐SeNPs and Nab‐PTX exerts synergistic efficacy, reduces toxicity, and promotes immunity in ESCC, providing a high‐efficiency and low‐toxicity therapeutic strategy (Huang et al. 2025)
SelenoM	Upregulated	NB, G	Regulates hypothalamic neuroendocrine; promotes glycolysis	Positively correlated with tumor metabolic reprogramming (*r* = 0.71) (Meng et al. 2025)
SelenoW	Upregulated	BRCA	SEPW1 deficiency activates the p53‐p21 pathway, blocks the G1/S phase of the cell cycle, dependent on p53 and p21	SEPW1 can be used as a therapeutic target to inhibit cancer cell proliferation (applicable to p53 wild‐type cancers) (Hawkes and Alkan 2011)
SelenoS	ER stress‐induced changes	PCA, BRCA, FSA	Promotes cancer cell apoptosis by activating the IRE1/ATF‐6 pro‐apoptotic pathway	MSA inhibits tumors by activating the SELENOS‐related apoptotic pathway (Goltyaev et al. 2020)
SelenoF	Downregulated, upregulated (CRC)	PCA, BRCA, CRC	In prostate cancer, SELENOF deficiency promotes the Warburg effect (enhanced glycolysis); in breast cancer, overexpression can inhibit the PI3K/AKT pathway and induce cell apoptosis	Low expression of SELENOF in prostate cancer and breast cancer can serve as a poor prognostic indicator (Flowers et al. 2023)
SelenoK	Membrane‐localized upregulation	MM, L	Calcium signal regulation; enhances PD‐1 response	The objective response rate (ORR) to anti‐PD‐1 therapy in patients with high expression reaches 45%; combination therapy prolongs survival by 2‐fold (Nease et al. 2024; Liu et al. 2024)
SelenoI	Upregulated	CRC, GC, RAC	SelenoI inhibits ferroptosis by maintaining ether lipid homeostasis (especially the balance of ether phospholipids ePE and ePC)	High expression of SELENOI in CRC promotes tumor growth (Huang et al. 2024)
SelenoO	Upregulated	MM	SELENOO promotes the survival and metastasis of cancer cells in the blood by AMPylation modification of mitochondrial protein (SDHA)	Can be used as a potential therapeutic target for melanoma and an indicator for predicting metastasis risk (Nascentes Melo et al. 2025)
SelenoT	Upregulated (HCC), downregulated (GC)	HCC, GC	In HCC, SELENOT performs redox regulation and inhibits apoptosis; in GC, it causes ER stress imbalance and decreased antioxidant capacity	SELENOT shows tissue‐specific expression, associated with cancer diagnosis, prognosis, and targeting (Zhao et al. 2015; Lan et al. 2017; Guariniello et al. 2015)
SelenoH	Significantly upregulated	CRC	SELENOH blocks G1/S phase transition by upregulating p21 and downregulating CCNE1, and promotes cancer cell differentiation, thereby inhibiting colorectal cancer proliferation	High expression of SELENOH is positively correlated with cell differentiation and may serve as a negative biomarker for the malignancy of CRC (Bertz et al. 2018)
SelenoV	Downregulated	GC	SELENOV has redox function, can scavenge ROS/RNS, enhance antioxidant capacity, and simultaneously inhibit ER stress and apoptotic pathways, improving mitochondrial function	Low expression of SELENOV is associated with poor prognosis of gastric cancer, its enhancement can inhibit cancer, and its antioxidant and anti‐ER stress mechanisms may be universally applicable to multiple cancer types (Zhang et al. 2020; Lan et al. 2017)
MSRB1	Upregulated	CRC, HCC	MSRB1 promotes CRC proliferation, invasion, and EMT (epithelial‐mesenchymal transition) by activating the GSK‐3β/β‐catenin signaling axis	Knockdown of MSRB1 significantly inhibits malignant tumor phenotypes, suggesting that it can be used as a new therapeutic target for CRC (Chen, Yang, et al. 2021; Li et al. 2018; He et al. 2018)
GPX1	Upregulated	CRC, HCC, PCA	Enhances tumor invasion and metastasis; inhibits cancer cell apoptosis; induces EMT (epithelial‐mesenchymal transition)	Inhibition of GPX1 can sensitize H_2_O_2_/cisplatin therapy (Ansong et al. 2014)
GPX2	Downregulated	BRCA	GPX2 affects angiogenesis and metabolic plasticity by regulating the ROS/HIF1α/VEGFA signaling axis, and is a core driver of breast cancer progression	Low expression of GPX2 is an independent poor prognostic factor for breast cancer (especially aggressive subtypes) (Ren et al. 2022)
GPX3	Upregulated	MM, BRCA	Eliminates reactive oxygen species (ROS) in lung tissue and reduces oxidative stress damage.	Serum exosomal miR‐21/29a or lung tissue GPX3^+^ AT2 cells can serve as early metastasis risk markers (Wang et al. 2022)
GPX4	Upregulated	BRCA, PCA, CRC, NB	Inhibits ferroptosis; degrades lipid peroxides, maintains a reducing environment dependent on the GSH‐Xc^−^ axis	RSL3 inhibitor induces ferroptosis; combination with immunotherapy enhances efficacy (DeAngelo et al. 2025; Gu et al. 2025)
GPX6	Upregulated	GC, BT	GPX6 reduces oxidative damage by scavenging reactive oxygen species (ROS) and lipid peroxides, and inhibits cancer cell proliferation and invasion	Targeting GPX6 may enhance antioxidant therapies (such as inducing ferroptosis), but its role in various cancer types needs further verification (Cueto‐Ureña et al. 2023; Ye et al. 2022)
TXNRD1	Upregulated	CRC, GC, HCC	Activates the NF‐κB pathway to promote survival; maintains the reduced state of thioredoxin	Enhances radiosensitivity (Yagublu et al. 2011)
TXNRD2	Upregulated (PCA), downregulated (BRCA)	PCA, BRCA	Inhibition of TXNRD2 leads to ROS accumulation causing cell death (TNBC); upregulation of TXNRD2 may enhance antioxidant capacity and promote cancer cell resistance to oxidative stress (CRPC)	Inhibition of TXNRD2 can induce cancer cell senescence and inhibit tumor growth; elevated serum TXNRD2 indicates castration‐resistant transformation and may be used for early prediction of drug resistance (Wang et al. 2025; Yang et al. 2020)
TXNRD3	Downregulated	CRC	Deficiency leads to calcium homeostasis disorder, inhibits pyroptosis (decreased NLRP3/Caspase‐1) and necrosis (decreased RIPK3/MLKL), thereby promoting carcinogenesis	Low selenium status increases risk; can be used as a diagnostic marker for IBD/CRC (Liu et al. 2022)
DIO1	Downregulated	THCA, RCC	In kidney cancer, DIO1 blocks proliferation by regulating cell cycle genes and inhibits metastasis by regulating adhesion and migration‐related molecules; its deficiency impairs thyroid hormone metabolism and antioxidant repair functions	Low expression of DIO1 is associated with poor prognosis of kidney cancer, restoration of its expression or targeted therapy is a potential strategy, and selenium supplementation needs to be evaluated individually (Arnaldi et al. 2005; Zhao et al. 2022; Poplawski et al. 2017)
DIO2	Upregulated	CRC, BLCA, BRCA	DIO2 catalyzes T4–T3 to activate TH signaling and promote tumor growth, acting through increasing vascular density, regulating the immune microenvironment, and the TGF‐β/EMT/Wnt pathway; overexpression is associated with low promoter methylation	High expression of DIO2 can indicate poor prognosis in cancer types such as CRC and BLCA; targeted inhibition of DIO2 and combination therapy can inhibit tumors (Nappi et al. 2025; Kojima et al. 2019)
DIO3	Downregulated (THCA), upregulated (HCC)	THCA, HCC	In THCA, DIO3 is silenced due to epigenetic modification, and demethylating agents combined with inhibitors can restore its expression; in HCC, Wnt pathway activation leads to high DIO3 expression, promoting cell cycle and inhibiting apoptosis through the pathway	ow expression of DIO3 in thyroid cancer can serve as a prognostic indicator; high expression of DIO3 in liver cancer is positively correlated with β‐catenin mutation and tumor progression (Alves et al. 2024; Sanceau et al. 2024)

Abbreviations: BLCA, bladder cancer; BRCA, breast cancer; BT, brain cancer; CRC, colorectal cancer; ESCC, esophageal squamous cell carcinoma; FSA, fibrosarcoma; G, glioma; GC, gastric cancer; HCC, liver cancer; L, lymphoma; MM, melanoma; NB, neuroblastoma; PCA, prostate cancer; RAC, rectal adenocarcinoma; THCA, thyroid adenoma.

Research has revealed that the selenoproteins GPX2 and GPX4 exert the most significant impact on the tumor microenvironment. Their influence manifests primarily in three aspects: immune cells, angiogenesis, and the extracellular matrix. For instance, GPX4 deficiency leads to lipid peroxide accumulation and ferroptosis in Treg cells, impairing their immunosuppressive function. This promotes CD8^+^ T cell infiltration and anti‐tumor immunity. Furthermore, GPX4‐deficient Treg cells can also increase IL‐1β secretion via mitochondrial superoxide accumulation, thereby promoting Th17 differentiation and breaking immune tolerance (Xu et al. 2021). Conversely, GPX2 deficiency upregulates VEGFA through the ROS/HIF1α axis, resulting in increased but structurally disorganized vascular density (Ren et al. 2022).

### 4.1 Selenocysteine (Sec)

#### 4.1.1 The Synthesis Mechanism of Selenocysteine

Selenocysteine, commonly abbreviated as “U” or “Sec,” is recognized as the 21st natural amino acid, featuring a distinct molecular structure and synthesis mechanism. Encoded by the stop codon UGA (Kryukov et al. 2003; Arnér 2010; Labunskyy et al. 2014), it represents a sulfur‐to‐selenium substitution variant of cysteine (Cys) (Reich and Hondal 2016; Maroney and Hondal 2018). Under physiological pH conditions, Sec demonstrates markedly different chemical properties from Cys. With a relatively low pKa of approximately 5.2, in contrast to Cys's pKa of 8.5, Sec mainly exists in an ionized state. This confers upon Sec a much higher nucleophilicity than Cys. Such enhanced nucleophilicity endows Sec with greater reactivity in chemical reactions, allowing it to engage more efficiently in a wide range of biochemical processes. Furthermore, Sec exhibits greater resistance to inactivation by diverse oxidants compared to Cys (Reich and Hondal 2016; Snider et al. 2013), Also, compared with its corresponding disulfide bond, Sec is capable of forming a substantially stronger diselenide bond (Arai et al. 2017). These unique physicochemical characteristics enable Sec to typically display higher activity than cysteine when participating in analogous reactions within biological systems.

The biosynthesis of Sec represents a highly complex and intricate process that takes place on specific tRNASec molecules. Initially, serine (Ser) is covalently linked to tRNASec by a specialized selenocysteine‐synthesizing machinery, yielding the Ser‐tRNASec molecule. Subsequently, through a cascade of enzymatic reactions, Ser undergoes conversion into Sec. This ultimately results in the formation of the Sec‐tRNASec molecule, which is precisely incorporated into selenoproteins at the UGA codon within a defined reading frame (Serrão et al. 2018). Significantly, Sec lacks its own dedicated aminoacyl‐tRNA synthetase (aaRS). Instead, it ingeniously harnesses the endogenous serine‐tRNA synthetase (SerRS) to execute the aminoacylation of serine. This distinctive mechanism vividly demonstrates the remarkable intermolecular cooperation within biological systems (Cain and Krahn 2024). Sec is extremely reactive and is readily auto‐oxidized by oxygen to form selenocystine. However, within cells, an essential reductase, thioredoxin reductase (TrxR), exists. TrxR can rapidly reduce selenocystine back to Sec, thereby establishing a unique redox cycle (Björnstedt et al. 1995). However, this cycle is not prevalent in biological systems because it persistently consumes NADPH while generating ROS (in the presence of oxygen). Therefore, to prevent excessive depletion of cellular resources and aggravation of oxidative stress, cells employ stringent regulatory mechanisms. These mechanisms ensure that Sec is either promptly incorporated into selenoproteins to fulfill its biological functions or catabolized by selenocysteine β‐lyase into L‐alanine and selenide. This process thereby maintains the homeostatic balance of Sec within the cell (Esaki et al. 1982).

In mammals, upon ingestion, diverse forms of dietary selenium embark on a series of intricate metabolic pathways, giving rise to a variety of intermediate metabolites. Among these, selenide (HSe), a pivotal intermediate, functions as the Se donor for the biosynthesis of Sec, furnishing the essential raw materials for Sec synthesis. Initially, selenium in the form of Sec was identified within the redox‐active glutathione peroxidase 1 (GPX1), precisely situated at the enzyme's active site. Subsequently, it became clear that the 21st amino acid, selenocysteine, is encoded by the stop codon UGA and incorporated into GPX. This represents an evolutionary‐conserved mechanism across all selenoproteins (Qian et al. 2019).

#### 4.1.2 Functional Studies of Selenocysteine

In humans, deficiency of the Selenoprotein P (SePP) receptor, LRP8 (LDL receptor‐related protein 8), leads to an inadequate supply of selenocysteine (Sec). This subsequently impairs the synthesis of the key anti‐ferroptosis selenoprotein, glutathione peroxidase 4 (GPX4), ultimately triggering ferroptosis. This represents a novel cancer therapeutic strategy. LRP8 is a critical factor protecting MYCN‐amplified neuroblastoma cells from ferroptosis. Consequently, selenocysteine (Sec) is indispensable for the translational synthesis of the anti‐ferroptosis selenoprotein GPX4. This dependency arises from the low expression of alternative selenium uptake pathways, such as the heterodimeric system Xc^−^, composed of SLC3A2 (also known as 4F2hc or CD98) and the specific subunit SLC7A11 (xCT) (Alborzinia et al. 2023). Notably, Jiang et al. constructed a selenocysteine‐containing heptameric peptide (H‐Arg‐Sec‐Gly‐Arg‐Asn‐Ala‐Gln‐OH). By mimicking the active site of the antioxidant enzyme glutathione peroxidase (GPX), this peptide offers new therapeutic avenues for acute organ injury or dysfunction caused by hepatic ischemia–reperfusion (I‐R) injury, thereby opening new perspectives for investigating the interaction between selenocysteine and disease (Jiang et al. 2016). In a study investigating the cytotoxicity of selenocysteine, Vozza et al. employed the MTS assay to assess the cytotoxicity of Sec and MSC (Se‐methylselenocysteine) on Caco‐2 human epithelial cells and HepG2 human hepatocytes at various test concentrations (25, 50, and 100 μM). The results demonstrated that natural Sec reduced Caco‐2 cell viability by ≥ 63% at concentrations of 50 and 100 μM, whereas MSC exhibited no cytotoxicity at any of the tested concentrations (Vozza et al. 2019). Clinically, agents containing selenocysteine (Sec) have demonstrated favorable anticancer efficacy. Compared to organic selenium compounds such as selenomethionine (SeMet) and selenomethylselenocysteine, as well as selenium‐containing inorganic agents like selenite, selenocysteine exhibits superior anticancer activity, with IC_50_ values ranging from 3.6 to 37.0 μM (Das et al. 2021).

### 4.2 Selenoprotein N (SELENON)

Selenoprotein N (SELENON), alternatively referred to as SEPN1, is a transmembrane protein situated within the endoplasmic reticulum (ER) (Castets et al. 2012), It holds the distinction of being the first selenoprotein identified in relation to human diseases, with a specific association to rigid spine muscular dystrophy congenita (Moghadaszadeh et al. 2001). A recent investigation has disclosed that SELENON can function as a calcium sensor through its calcium‐binding EF‐hand domain. It activates the sarco/endoplasmic reticulum calcium ATPase (SERCA2) in a redox‐dependent fashion, thereby facilitating the uptake of calcium into the endoplasmic reticulum (Chernorudskiy et al. 2020). This discovery has opened up the possibility for the utilization of selenoproteins in the treatment of human diseases.

### 4.3 Selenoprotein P (SELENOP)

Selenoprotein P (SELENOP) is often referred to as a “selenium transport” which is distinctive in that it represents a secreted glycoprotein harboring as many as 10 Sec residues and contributes to 50% of the total selenium content within plasma. It exhibits diverse functions, including heavy‐metal‐binding capabilities and potential enzymatic redox activities (Schomburg 2022). Principally synthesized by the liver and secreted into the plasma, SELENOP plays a pivotal role in furnishing selenium to other selenium‐deficient tissues for the synthesis of other selenoproteins via blood‐borne transportation. Significantly, SELENOP's functionality is not confined to the liver; it is expressed in various tissues such as the testis, muscle, kidney, brain, small intestine, and colon (Barrett et al. 2015).

SELENOP encompasses two domains. The larger N‐terminal domain contains 1 Sec residue within the UXXC redox motif, while the C‐terminal domain houses 9 Sec residues and serves as a Se supplier. In the N‐terminal domain, SELENOP features two histidine (H)‐rich sequences, which dictate its heparin‐binding characteristics. When researchers probed into the association between the antioxidant function of selenium and diseases, they discovered that augmented levels of reactive oxygen species are intimately linked to the pathological progressions of Alzheimer's disease and Parkinson's disease. Selenoprotein P, endowed with antioxidant properties, can exert an inhibitory effect on these two diseases (Solovyev et al. 2018).

### 4.4 Selenoprotein K (SELENOK)

Selenoprotein K (SELENOK) is a small molecular weight protein (approximately 12 kDa) featuring a single transmembrane helix. Its N‐terminal sequence extends into the endoplasmic reticulum (ER) lumen, while its C‐terminus resides in the cytosol. The intrinsically disordered nature of SELENOK suggests its function relies on interacting partner proteins (Polo et al. 2016). The role of intrinsically disordered domains within proteins, serving as signaling molecules or docking platforms for binding proteins, has been demonstrated in other non‐enzymatic proteins (Simister and Feller 2012). Regarding immune cell function, SELENOK is indispensable for Ca2^+^ flux‐mediated migration in T cells and neutrophils, as well as for the migration and phagocytic activity of macrophages and microglia.

Furthermore, in the domain of oncology research, existing studies have documented anti‐cancer properties of selenoprotein K in in vivo melanoma models and human melanoma cell lines. While, the precise anti‐cancer molecular mechanisms remain to be explored in greater depth, it is evident that selenoprotein K has potential applications (Marciel and Hoffmann 2019).

### 4.5 Selenoprotein S (SELENOS)

Selenoprotein S (SELENOS), was first identified in the liver of the fat sand rat ( *P. obesus* ) by Walder et al. (2002) and initially designated as Tanis. Subsequent investigations, however, have demonstrated that Tanis, AD‐015, SelS, SELENOS, VIMP, and SEPS1 are, in fact, the same protein (Ye et al. 2004; Gao et al. 2006).

In the research by Walder et al. focusing on the connection between SELENOS and the inflammatory mechanism, it was revealed that SELENOS serves as the receptor for serum amyloid A (SAA), an acute‐phase inflammatory response protein. Notably, when the expression of SELENOS is inhibited, the expression of SAA in lipopolysaccharide (LPS)‐induced HepG2 human hepatocellular carcinoma cells is upregulated correspondingly. These findings imply a potential link between SELENOS and the inflammatory response (Walder et al. 2002). Subsequently, the study by Fradejas et al. further disclosed that the expression level of SELENOS increases in the brain tissue of C57BL/6 mice following inflammatory injury, reaffirming the correlation between SELENOS and inflammation (Fradejas et al. 2011). Additionally, induction of SELENOS overexpression, can reduce the expression of the inflammatory cytokines interleukin‐1β (IL‐1β) and interleukin‐6 (IL‐6) in LPS‐stimulated astrocytes. Conversely, the inhibition of SELENOS expression further elevates the expression levels of IL‐1β and IL‐6 under LPS stimulation. These results, to a certain extent, elucidate the molecular mechanism underlying the anti‐inflammatory function of SELENOS (Yu and Du 2017).

Selenoprotein S, an ER membrane protein, also plays an important role in maintaining intracellular ER morphology and distribution (Noda et al. 2014). Research by Kelly and Kim has indicated that the overexpression of SELENOS can decrease the activity of the glucose‐regulated protein 78 (GRP78) promoter (a marker protein for ER stress) in HepG2 hepatocellular carcinoma cells and also reduce the expression of GRP78 protein induced by thapsigargin (TC) in HEK293T human embryonic kidney cells (Kelly et al. 2009; Kim and Kim 2013).

In summary, SELENOS manifests multiple significant functions in areas such as inflammatory response and regulation of the physiological functions of the cell endoplasmic reticulum. Its modulation of inflammatory cytokines and interaction with ER stress‐related proteins offer crucial insights for a more in‐depth exploration of cell physiological and pathological processes. Additional research is warranted to comprehensively understand its functions and action mechanisms under diverse physiological and pathological conditions, thereby providing a more robust theoretical foundation for the treatment of related diseases and the development of drugs.

### 4.6 Selenoprotein W (SELENOW)

Selenoprotein W (SELENOW), a selenoprotein prominently expressed in the liver, occupies a crucial position within the antioxidant system. Initially, it was reported to be associated with the white appearance in selenium‐deficient regions of calcific cardiomyopathy (Whanger 2000). In 2021, Kim et al. analyzed the mRNA expression profiles during the large‐scale differentiation of osteoclasts induced by receptor activator of nuclear factor kappa‐B ligand (RANKL). They found that SELENOW is a protein whose expression is downregulated and is regulated by the RANKL/RANK/tumor necrosis factor receptor‐associated factor 6/p38 signaling pathway (Kim, Lee, et al. 2021). This research demonstrated that upon overexpression, SELENOW promotes the in vitro formation of osteoclasts via the nuclear translocation of NF‐κB and the nuclear factor of activated T cells cytoplasmic 1, which is mediated by 14‐3‐3γ. Conversely, the deficiency of SELENOW inhibits this process. During the investigation of non‐alcoholic fatty liver disease, Zhiruo Miao et al. discovered that SELENOW can trigger the transactivation of HIF‐1α by modulating the nuclear translocation of PKM2. This leads to mitochondrial apoptosis, ultimately resulting in mitochondrial damage, excessive production of reactive oxygen species (ROS), and leakage of mitochondrial DNA. The accumulation of mitochondrial ROS can further activate the NLRP3 inflammasome‐mediated apoptosis and facilitate the extracellular leakage of mtDNA. The leaked mtDNA can activate the cGAS‐STING signaling pathway in macrophages, thereby inducing a phenotypic transformation of macrophages (Miao et al. 2024).

These studies have shown that further elucidation of the mechanism of action of SELENOW on immune cells is one of the important directions in the research of selenoproteins. Determination of this mechanism of action is expected to provide a new theoretical basis and potential targets for the prevention and treatment of related diseases.

### 4.7 Selenoprotein M (SELENOM)

Selenoprotein M (SELENOM) is an ER‐resident redox enzyme exhibiting structural resemblance to thioredoxin (TXN). This protein is highly expressed in the hypothalamic regions engaged in leptin signaling. Notably, prior research has already associated it with energy metabolism (Gong et al. 2021; Pitts et al. 2013). Ting Gong et al. conducted parallel microarray analyses on hypothalamic tissues and mHypoE‐44 cells. Their findings revealed that the deficiency of SELENOM significantly impacts multiple genes (Gong et al. 2021).

### 4.8 GPX4

Glutathione peroxidase (GPX) is a member of the antioxidant enzyme family. Its catalytic site contains cysteine, a redox‐active residue (Flohé et al. 2022). In humans, five Sec‐containing GPX enzymes are currently known: the ubiquitously expressed cytosolic GPX (GPX 1), the gastrointestinal‐specific GPX (GPX 2), plasma GPX (GPX 3), the ubiquitously expressed phospholipid hydroperoxide GPX (GPX 4), and the olfactory epithelium‐ and embryonic tissue‐specific GPX (GPX 6) (Kryukov et al. 2003). In the mammalian enzyme system, GPX1‐4 are classified as selenium‐dependent enzymes, with their activities contingent upon the presence of selenium, while GPX6 is a selenoprotein. Among them, GPX4 has garnered significant attention owing to its crucial physiological functions and unique research value. Initially named phospholipid hydroperoxide glutathione peroxidase (PHGPX), it was successfully purified from porcine liver or heart tissue by Ursini et al. as early as 1982 (Ursini et al. 1982).

Functioning as a peroxidation‐suppressing protein on liposomes and biological membranes, GPX4 exhibits remarkable antioxidant capabilities. It can specifically identify and act on diverse oxidized substrates, including common reactive oxygen species such as organic peroxides, hydroperoxides, and hydrogen peroxide. Through catalytic reduction reactions, GPX4 converts these harmful substances into relatively benign products, thereby effectively safeguarding cells from oxidative damage and maintaining the stability of the intracellular milieu (Weaver and Skouta 2022). GPX4 is expressed in a wide range of tissues, with an especially high abundance in the testis, exerting a substantial influence on sperm development and function (Roveri et al. 2002).

**FIGURE 2:**
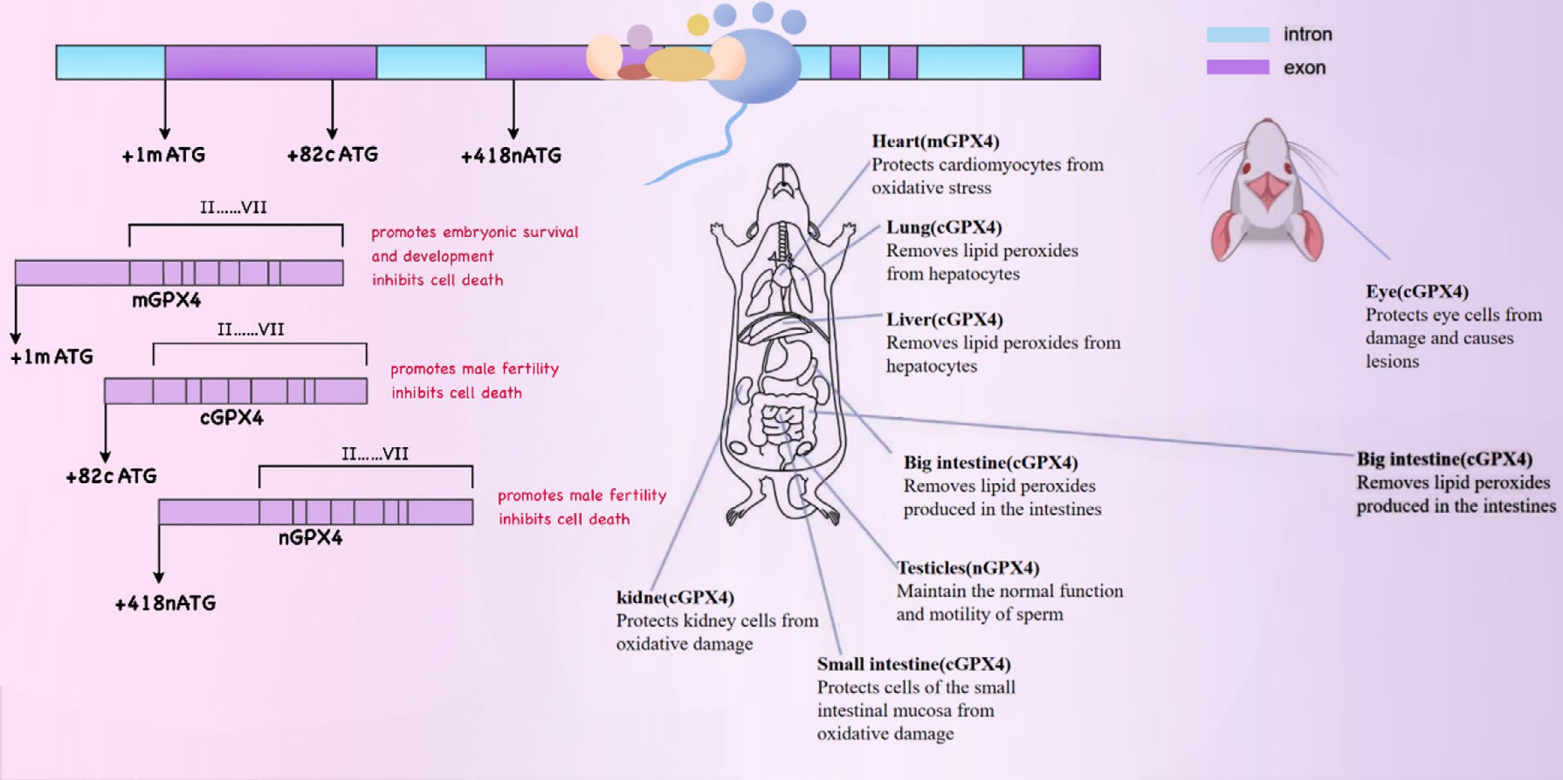
The effects of GPX4 members with different subcellular locations and spatial structures on the distribution and function of the members in animals.

GPX4 exists in three physiological subtypes: cytoplasmic (cGPX4), mitochondrial (mGPX4), and nuclear (nGPX4). Each subtype plays a distinct and pivotal role in different intracellular compartments. Specifically, nGPX4 is of great significance for ensuring the structural integrity of human sperm chromatin. By maintaining the compact structure and stability of chromatin, it guarantees the integrity and proper transmission of sperm genetic material. cGPX4 is an essential factor in sperm maturation and embryonic development, participating in multiple critical stages of spermatocyte differentiation, maturation, and early embryonic development (Brigelius‐Flohé 2006). Research has demonstrated that in the absence of GPX4, when transgenic mice expressing mGPX4 are crossed with those expressing cGPX4, only the mice expressing cGPX4 survive post‐embryonic development. This finding underscores the importance of cGPX4 in maintaining cellular redox homeostasis, integrity, and viability (Liang et al. 2009), Given the pivotal role of cGPX4 in maintaining cell survival and normal cell functions, its mechanism of action has emerged as a focal point in scientific research. A plethora of studies have indicated that cGPX4 is closely associated with the pathogenesis and progression of various diseases, such as neurodegenerative disorders (Chen et al. 2015), chronic obstructive pulmonary disease (COPD) (Yoshida et al. 2019), immune system disorders (Altamura et al. 2020), and cancer (Soula et al. 2020), among others. On the other hand, mGPX4 is predominantly localized in mitochondria, functioning within the core region of cellular energy metabolism. By restricting the accumulation of mitochondrial reactive oxygen species (ROS) or oxidized α‐ketoisocaproic acid (a metabolic intermediate in the tricarboxylic acid/tricarboxylic acid cycle), mGPX4 can effectively prevent oxidative‐stress‐induced cell death, preserve normal mitochondrial function, and maintain cell viability. This property renders mGPX4 a key component of mitochondrial anti‐apoptotic proteins and a structural constituent of sperm, making it indispensable for male fertility. Besides its role in sperm maturation, mGPX4 is also involved in the development and maturation of photoreceptors, although its precise mechanism remains to be fully elucidated. Additionally, research reports suggest that mGPX4 may play a role in inhibiting the increase in ferritin levels in cancer‐like cells, indicating its potential cytoprotective effect (Ursini et al. 1999; Azuma et al. 2022; Xavier et al. 2022; Liu, Wan, et al. 2023). As a multifunctional protein, GPX4 has attracted extensive attention in the fields of oncology, cardiovascular research, and neuroscience over the past decade. Unraveling the mechanisms that regulate GPX4 activity has thus become an important research area. The isoforms and in vivo distribution of GPX4 in mice are shown in Figure 2. The distribution and functions of different selenoproteins in the body vary, as shown in Table 1.

## 5 Role of Selenium in Immune Mechanisms

Among the known functions of selenium compounds in the immune mechanism, their anti‐inflammatory, antioxidant, and immune cell functions are the most prominent in various experiments and studies. At the same time, the study of selenium‐containing compounds, selenoproteins, on the mechanism of immune cells has emerged as a promising avenue of exploration.

### 5.1 Mechanisms of Selenium in the Anti‐Inflammatory Response

When tissues are afflicted by infection, poisoning, or mechanical trauma, damage‐associated molecular patterns (DAMPs) released by dead and dying cells, together with pathogen‐associated molecular patterns (PAMPs) produced by invading organisms, synergistically initiate an inflammatory response (Zhang et al. 2010). Inflammation represents a fundamental defense reaction against harmful stimuli. It serves to eliminate invading pathogens and foreign materials, thereby restoring homeostasis. Nevertheless, over‐activation of the inflammatory response can inflict harm on the host, giving rise to conditions such as cancer, sepsis, and autoimmune diseases. This is accompanied by clinical manifestations like fever, pain, erythema, and swelling, typically induced by infection and tissue injury (Sun et al. 2020).

Selenium plays a pivotal role in stabilizing and regulating human metabolism within the human body and is of significant importance for its anti‐inflammatory properties. For instance, during the wound‐healing process, selenoproteins such as GPX1, GPX4, selenoprotein S, and selenoprotein P engage in diverse reactions during the inflammatory phase. These include antioxidant activities, the inhibition of inflammatory cytokines, and the elimination of peroxynitrite (a super‐radical ion), functioning as antioxidants and inducers at specific stages.

Upon wound occurrence, platelets aggregate to form a thrombus, and immune cells release pro‐inflammatory cytokines and neutrophils, generating substantial amounts of enzymes involved in oxidative stress regulation (associated with ROS production), such as catalase (HP) and lipid peroxidase (LP) (Hariharan and Dharmaraj 2020). ROS are a class of chemical species generated following the incomplete reduction of molecular oxygen. They primarily encompass hydroxyl radicals (·OH), superoxide anions (O_2_^−^), singlet oxygen (^1^O_2_), and hydrogen peroxide (H_2_O_2_), and play crucial roles in cell signaling and homeostasis. In the context of anti‐inflammatory research progress, it has been revealed that the pathogenesis of various health disorders, including neuro‐degenerative diseases and cancer, is attributed to the accumulation of ROS. This accumulation leads to oxidative stress and inflammation (Leiter et al. 2022; Rusu et al. 2022). At moderate levels, ROS act as key signaling molecules, regulating diverse physiological functions, including the inflammatory response. However, when present in excessive amounts, ROS exert toxic effects, directly oxidizing biological macromolecules such as proteins, nucleic acids, and lipids. This exacerbates the progression of the inflammatory response and precipitates diseases (Liu, Han, et al. 2023). In the presence of environmental stress, ROS levels surge rapidly, thereby triggering oxidative stress.

Glutathione peroxidase (GPX) is capable of effectively alleviating inflammatory diseases induced by excessive reactive oxygen species (ROS) during the body's inflammatory response. GPX reduces hydrogen peroxide to water and converts organic hydroperoxides (ROOH) into alcohols (ROH) according to the reaction: ROOH + 2GSH → ROH + H_2_O + GSSG. This process effectively curtails free‐radical reactions to a tolerable level (Kieliszek and Błażejak 2016). During the inflammatory process, GPX can transform hydrogen peroxide into peroxidase, thereby circumventing the Fenton reaction and the Haber‐Weiss reaction (Certain metals possess unique oxygen‐transfer properties that enhance the utilization of hydrogen peroxide, while others exhibit strong catalytic abilities, generating highly reactive hydroxyl radicals.) As a result, GPX efficiently scavenges free radicals. Selenium‐dependent GPX1‐4 and GPX6 directly suppress oxidative stress. Notably, GPX1 represents the most abundant and the first‐identified selenoprotein in mammals (Avery and Hoffmann 2018), and the main metabolic form of selenium in the body to cope with severe oxidative stress (Bjørklund et al. 2022), The GPX family plays a crucial role in safeguarding the body's antioxidant defense system.

### 5.2 Mechanisms of Selenium in Antioxidant Response

Selenium (Se), as an essential dietary trace element, is closely associated with the antioxidant functions of selenoproteins. It plays a critical role in reducing oxygen‐derived oxidative metabolites and mitigating inflammatory responses in the endothelium (Benstoem et al. 2015; Fakhrolmobasheri et al. 2022). During cardiovascular surgery, higher serum selenium levels have been shown to correlate with myocardial protection following ischemia–reperfusion injury (Mazaheri‐Tehrani et al. 2024). Selenium is recognized as a natural antioxidant owing to its intrinsic antioxidant characteristics. Considering that cancer cells are particularly vulnerable to reactive oxygen species (ROS), targeting the antioxidant susceptibility of tumor cells has emerged as an extremely promising anti‐cancer strategy (Cairns et al. 2011). Unlike other anti‐cancer therapies during the anti‐cancer oxidation process, selenium functions as a pro‐oxidant to impede the growth of cancer cells while remaining devoid of side effects on normal cells. This property of selenium is beneficial in alleviating the human toxicity of anti‐cancer drugs (Huang et al. 2012; Weekley and Harris 2013). Oxidative stress induced by free radicals in living organisms is intimately linked to the development of malignant tumors. Chemotherapeutic agents, radiotherapy, and ionizing radiation all contribute to the generation of free radicals and the induction of cytotoxicity (Greenberger et al. 2001; Girdhani et al. 2005). For example, an excess of ROS can modify mitochondrial potential and trigger the mitochondrial‐dependent apoptotic signaling pathway (Addabbo et al. 2009). Research by Xia et al. indicates that selenium can modulate immune function by inhibiting nuclear factor NF‐κB, regulating the Nrf2 transcription factor, and influencing ferroptosis (Xia et al. 2021). These functions are primarily associated with the mechanism by which selenium metabolites undergo redox conversion into simple compounds possessing potent protective and antioxidant properties (Kim, Choi, et al. 2021). Selenoproteins demonstrate considerable potential in preventing the progression of Alzheimer's disease. One of the most significant biological functions of selenoproteins is their antioxidant activity against oxidative stress and their capacity for reversible regulation, mediated through redox proteins (Hariharan and Dharmaraj 2020). Selenoproteins such as TXNRD and MSRB1 play crucial roles in regulating redox activity and repairing immune cells damaged by oxidative stress (Razaghi et al. 2021). These properties confer strong potential to selenium for preventing the accumulation of β‐amyloid protein, a misfolded protein produced during inflammation associated with Alzheimer's disease. This also provides evidence supporting the potential application of selenium in the treatment of amyloidosis (Kieliszek and Sapazhenkava 2025). Consequently, investigating the antioxidant effects of selenoproteins is essential for understanding the role of selenium in cancer prevention and therapy.

### 5.3 Mechanisms of Selenium in Innate Immunity

Selenium represents a crucial element in the regulation of the immune systems of both humans and animals. It participates in immune modulation via inorganic and organic forms of selenium, with selenoproteins being the primary organic form (Jia et al. 2021). Specifically, selenium compounds regulate innate immunity mainly by regulating the activities of immune cells such as dendritic cells, NK cells, and neutrophils, thereby exerting immunomodulatory effects in the human body. Innate immunity constitutes the first line of defense for the host against danger signals and functions in a rapid and non‐specific fashion. Innate immune cells activated by selenium compounds can promote tissue inflammation through the secretion of pro‐inflammatory mediators, including tumor necrosis factor‐α (TNF‐α), interleukin‐6 (IL‐6), and reactive oxygen species (ROS) (Yang, Zhang, et al. 2014).

#### 5.3.1 Selenium and Granulocytes (PMN)

Granulocytes (PMN), encompassing neutrophils, eosinophils, basophils, and mast cells, mediate innate immunity through their involvement in inflammation and phagocytosis (Vorobjeva et al. 2023). Their remarkable bactericidal capacity is, in part, ascribable to their ability to generate substantial amounts of intracellular and extracellular reactive oxygen species (ROS) via the activation of membrane‐associated nicotinamide adenine dinucleotide phosphate (NADPH) oxidase 2 (NOX 2) (Winterbourn et al. 2016; Zeng et al. 2019). Several studies have shown that selenium deficiency leads to aberrant PMN pro‐inflammatory functions, including inhibition of ROS production (Arthur et al. 2003), effects on bactericidal capacity (Lee et al. 2022) and alteration of Netosis (the mode of inflammatory cell death of neutrophils) (Chi et al. 2021; Zhou et al. 2021).

Numerous proteins have been identified as directly implicated in regulating PMN functions. For instance, the lipid peroxidation regulator GPX4 has been found to modulate PMN ferroptosis in lupus patients (Li et al. 2021), The deletion of selenoprotein K in mouse PMN reveals that, upon stimulation by KC (also referred to as CXCL1), Ca^2+^ influx and PMN migratory capacity are diminished (Verma et al. 2011), In female animals, PMN lacking the expression of thyroid hormone deiodinase 3 (DIO3) exhibit impaired NOX enzymatic activity (van der Spek et al. 2018). During phagocytic activities, neutrophils generate abundant ROS, such as superoxide and H_2_O_2_, through NADPH oxidase, primarily NOX2, thereby triggering an oxidative burst. This process confers upon neutrophils potent antibacterial capability (Babior et al. 2002).

Excessively high levels of ROS can be detrimental to neutrophils themselves. Consequently, neutrophils possess an essential self‐protection mechanism. Research has shown that following the completion of critical immune defense functions, neutrophils migrate from the site of sterile inflammation to the lungs and then re‐direct to the bone marrow, ultimately concluding their immune cycle and withdrawing from circulation (Silvestre‐Roig et al. 2020). Human PMN treated with TNF‐α, a pro‐inflammatory cytokine, indicates that selenoproteins mediate the self‐protection of neutrophils against oxidative damage (Hattori et al. 2005), These findings substantiate the pivotal role of selenoproteins in neutrophil physiology.

#### 5.3.2 Selenium and Dendritic Cells (DCs)

The impacts of selenium on dendritic cells (DCs) are manifold. Adequate selenium supplementation facilitates the maintenance of a balance between the phagocytic capacity and the migratory potential of immature DCs. It also promotes the chemotactic migration of mature DCs. Selenium modulates DC subsets. Reportedly, selenium supplements are capable of reducing the proportion of activated DCs while augmenting the number of tolerogenic DCs (Huang et al. 2021; de Toledo et al. 2020). As a class of specific antigen‐presenting cells consisting of diverse subsets, DCs exhibit a robust ability to capture and process antigens and play a pivotal role in both innate and adaptive immune processes (Worbs et al. 2017). Jia et al. demonstrated that selenium at varying concentrations can regulate the differentiation of dendritic cells. They isolated monocytes and DC‐monocytes from human peripheral blood and determined the expression levels of selenoproteins in immature DCs (imDCs) and mature DCs (mDCs) using techniques such as inductively coupled plasma mass spectrometry. The findings revealed that GPX1 and GPX4 were the most abundantly expressed, whereas the expression levels of DIO and GPX6 were comparatively low. Further investigations indicated that in imDCs, a selenium concentration of 0.1 μM enhanced their anti‐phagocytic activity, while a concentration of 0.2 μM inhibited this activity. Regarding cell migration, 0.1 μM of selenium promoted the migration of both imDCs and mDCs, yet 0.05 or 0.2 μM of selenium impeded this process. Additionally, in the mixed lymphocyte reaction, 0.1 μM of selenium ameliorated the reaction, while 0.05 and 0.2 μM of selenium inhibited it (Jia et al. 2021).

Furthermore, there exists a close association between dendritic cells and the SELENOK protein. Huan Xia et al. generated imDCs from male wild‐type (WT) mice and SELENOK knockout (KO) mice and conducted in‐depth investigations into their immune functions. Simultaneously, they examined the effects of the endoplasmic reticulum stress (ERS) inducer tunicamycin (Tm) on the migration, phagocytosis, and phenotypic markers of WT imDCs. Results indicated that in Tm‐treated imDCs, both the gene expression level and protein content of SELENOK increased gradually. This fully demonstrates that SELENOK plays a crucial role in the immune function of dendritic cells and is indispensable for the proper manifestation of their immune functions (Xia et al. 2022).

#### 5.3.3 Selenium and Macrophages (BMDM)

Macrophages, an integral part of the human immune system, exhibit a wide array of physiological functions. These encompass coordinating innate and adaptive immune responses, eliminating cellular debris, and orchestrating tissue development and homeostasis (Mosser and Edwards 2008; Wynn et al. 2013). As macrophages engage in these physiological processes, they generate diverse cytokines in response to various stimuli, such as tissue injury and microbial infections. These cytokines assume multifaceted roles within the immune response. Some of them can promote the inflammatory reaction and activate immune effector mechanisms at specific sites, thereby bolstering the body's capacity to eliminate pathogens. Notably, among numerous macrophage‐related proteins, selenium‐containing MSRB1 is highly expressed in macrophages under immune‐activated states. A recent study, employing rigorous experimental approaches, has demonstrated that MSRB1 plays a pivotal role in the process by which lipopolysaccharide (LPS)‐stimulated macrophages produce anti‐inflammatory factors. It is a critical determinant in inducing macrophages to generate two key anti‐inflammatory factors, interleukin‐10 (IL‐10) and interleukin‐1 receptor antagonist (IL‐1RA) (Lee et al. 2017).

Sougat Misra and co‐workers carried out an in‐depth investigation employing gene‐knockout (KO) technology to establish a mouse bone‐marrow‐derived macrophage (BMDM) model with the aim of exploring the mechanism of action of SELENOW in the inflammatory process triggered by the bacterial endotoxin lipopolysaccharide (LPS). The research findings revealed that under low‐selenium conditions (low‐selenium −/− bone marrow stromal cells), the expression level of arginase‐1 was markedly decreased. Arginase‐1, a key enzyme closely linked to the anti‐inflammatory (M2) phenotype, plays an indispensable role in the resolution of inflammation. These results strongly indicate that the expression of SELENOW in macrophages is of great significance for regulating cellular redox processes and overall biological behaviors during the onset and resolution of inflammation, further underscoring the crucial role of selenoproteins in the immune function of macrophages (Misra et al. 2023).

### 5.4 Mechanisms of Selenium in Adaptive Immunity

T lymphocytes and B lymphocytes serve as the central cells of adaptive immunity. Selenium and its compounds participate in the composition of numerous selenoproteins, including GPXs, SELENOP, SELENOK, and others. In selenium‐deficient individuals, the translation of selenoproteins ceases at the selenocysteine (Sec) encoded by the UGA codon. Subsequently, the mRNA undergoes nonsense‐mediated decay, and the truncated proteins are degraded via C‐terminal degradation. During this process, the brain, muscles, and testes are preferentially supplied with bioavailable selenium, while other tissues, such as those that make up the immune system, suffer impairment (Lin et al. 2018; Seyedali and Berry 2014), This ultimately results in a decline in adaptive immune function. Adequate selenium supplementation, however, can counteract this deleterious process.

#### 5.4.1 Selenium and T and B Lymphocytes

T lymphocytes play a pivotal role in orchestrating adaptive immunity against pathogens and cancer, as well as in regulating immune tolerance. Specifically, CD4^+^ T cells serve as crucial regulators within the adaptive immune system. They assist CD8^+^ T cells and B cells, thereby coordinating the overall immune response. Naïve T cells mature and circulate throughout the body. Upon encountering antigen‐presenting cells (APCs), their T‐cell receptors (TCRs) bind to antigens, triggering T‐cell activation (Ma and Hoffmann 2021). Following activation, T cells initiate a signaling cascade that promotes the transcription of IL‐2 mRNA. IL‐2, acting as the primary growth factor for T cells, drives their growth and proliferation. Similarly, the activation of CD8^+^ T cells also depends on IL‐2 (Cui and Kaech 2010). The signaling networks involved in T‐cell activation are under stringent regulation and are intricately associated with alterations in cell metabolism.

**FIGURE 3:**
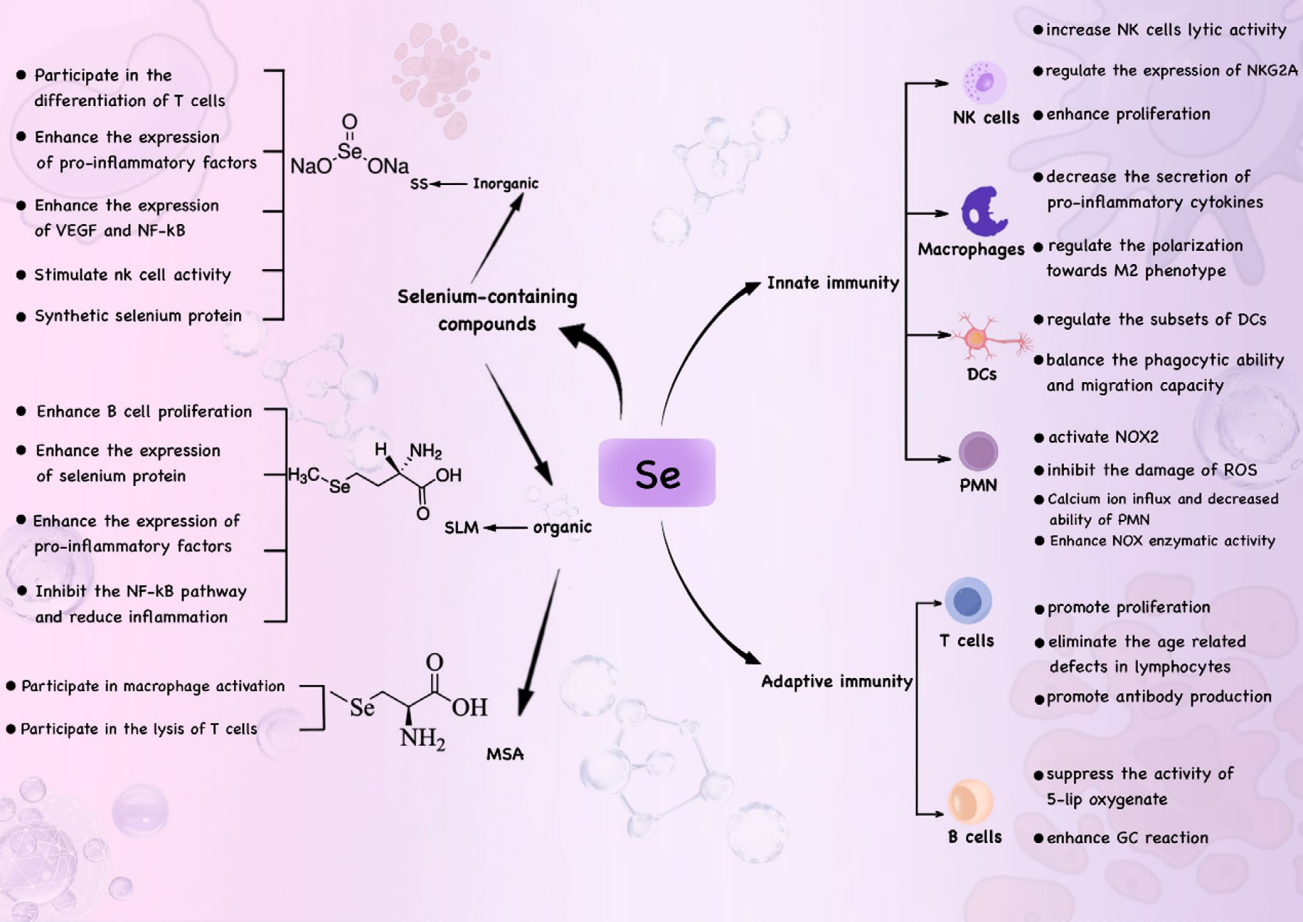
Selenium‐containing compounds of inorganic and organic nature regulate the viability of NK cells, macrophages, DCs, granulocytes, mast cells, and microglia in innate immunity and affect the proliferation and differentiation of T and B cells in adaptive immunity.

Selenium enhances the expression of the α (p55) and/or β (p70/75) subunits of the growth‐regulatory lymphokine interleukin‐2 receptor (IL‐2R). Through this action, selenium promotes the interaction between the α and/or β subunits of IL‐2R and interleukin‐2, ultimately accelerating the rate of cell proliferation and enhancing the rate of differentiation into cytotoxic cells, thereby manifesting its corresponding functions (Zhang et al. 2023) (Figure 3). A study on human subjects with compound heterozygous defects in the SECISBP2 gene, which reduces the synthesis of 25 known human selenoproteins, demonstrated impaired T cell proliferation and abnormal cytokine secretion by peripheral blood mononuclear cells (Stoupa et al. 2024). This indicates that selenoproteins are indispensable for T cell‐mediated immune functions in humans. In a randomized clinical trial conducted by Dehghani et al. the effects of 3 months of selenium intake on the frequency of CD4^+^CD25^+^FOXP3^+^ Treg cells and immune checkpoint receptor expression were investigated in patients with diffuse large B‐cell lymphoma (DLBCL) subtype non‐Hodgkin lymphoma (NHL) 16 patients receiving Se (Se^+^) and 16 patients not receiving Se (Se^−^). Flow cytometry and SYBR Green real‐time PCR were employed to evaluate changes in Treg cell frequency and the expression of immune checkpoint receptors (including CTLA‐4, LAG‐3, TIM‐3, and PD‐L1 genes) in the two groups (Dehghani et al. 2021). The results of this study suggest that selenium may influence the functional characteristics of CD4^+^CD25^−^FOXP3^+^ T cells, thereby impacting adaptive immune function.

## 6 Selenium Metabolism in Solid Tumors

### 6.1 Overview of Selenium Metabolism

**FIGURE 4:**
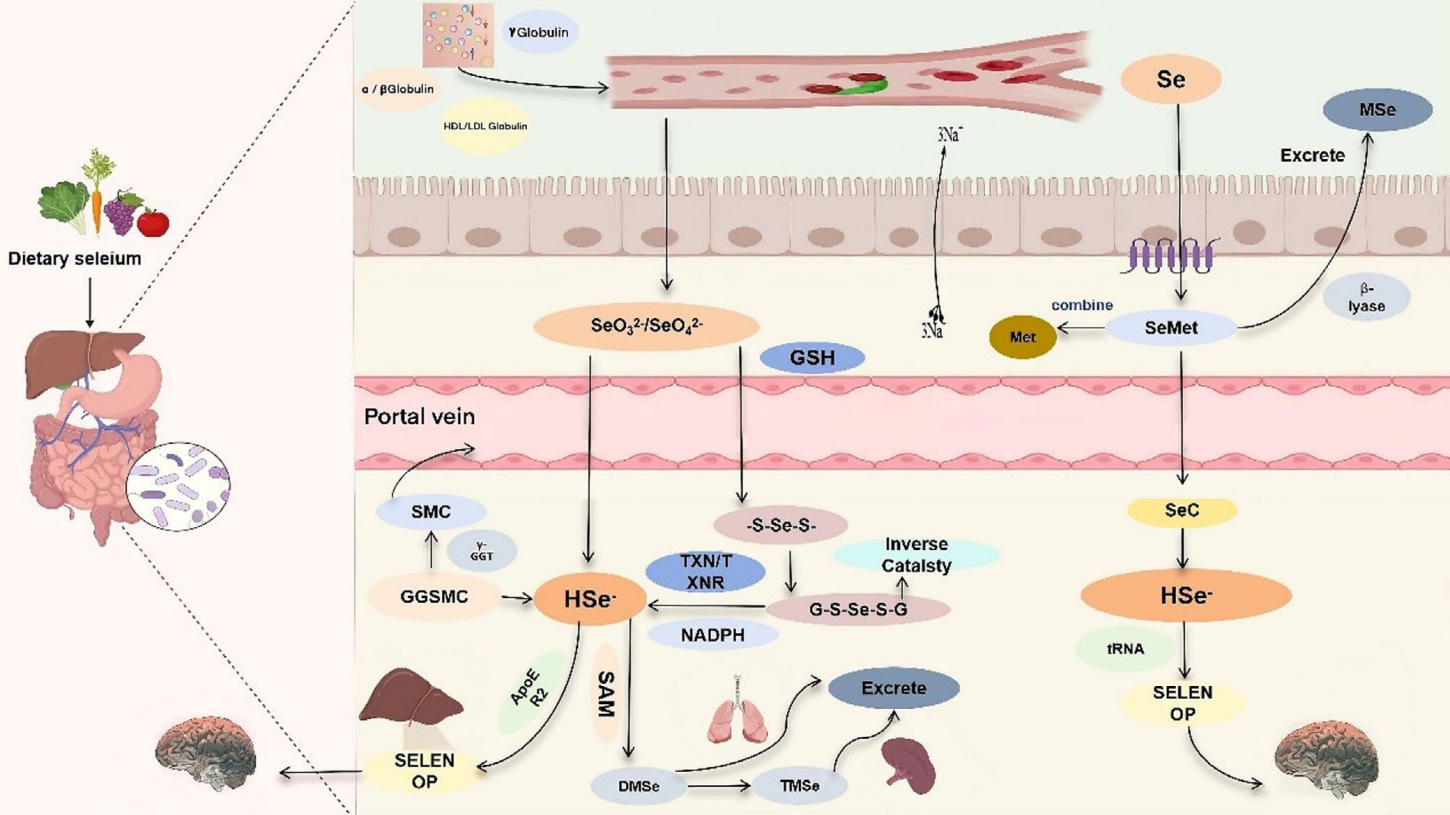
In mammals, absorbed and assimilated selenium generates hydrogen selenide (HSe^−^), which is utilized in the synthesis of selenoproteins. These selenoproteins are subsequently transported to the brain. Ultimately, the methylated metabolites of selenium are excreted via pulmonary or renal pathways.

Dietary selenium, encompassing inorganic selenate (SeO₄^2−^) and selenite (SeO₃^2−^), as well as organic selenomethionine (SeMet), is absorbed via the gastrointestinal tract. Following absorption, it is transported within the body via α/β‐globulins, HDL/LDL lipoproteins, and γ‐globulins, entering the systemic circulation through the portal vein. The metabolic pathways of selenium exhibit significant differences depending on whether it exists in inorganic or organic forms. Inorganic forms, such as selenite and selenate, are reduced to hydrogen selenide (HSe^−^) in the presence of glutathione (GSH), catalyzed by the enzyme thioredoxin reductase (TXNRD). This HSe^−^ subsequently reacts to form selenodiglutathione (GSSeSG). Glutathione reductase then converts GSSeSG into glutathione selenol (GSSeH). Finally, GSSeH is decomposed back to HSe^−^ by glutaredoxin (Newton and Pluth 2019). As organic selenium‐containing compounds, SeMet and Sec are assimilated via transcellular pathways and share transporters with their sulfur‐containing analogues (Nickel et al. 2009). Selenomethionine (SeMet) can be assimilated via a Na^+^‐dependent process or nonspecifically incorporated into proteins at methionine (Met) residues. Alternatively, it can be converted to selenocysteine (Sec), which is subsequently transformed into selenide (HSe^−^) through the trans‐selenation pathway (Suzuki and Ogra 2002; Roman et al. 2014). Sec, another selenoamino acid released during the catabolism of selenoproteins, is recycled back to HSe^−^ for selenium utilization or excretion. Selenium‐methylselenocysteine (SMC) and the seleno‐dipeptide γ‐glutamyl‐Se‐methylselenocysteine (GGSeMSC) are assimilated in the gastrointestinal tract. Here, the majority of GGSeMSC is hydrolyzed by γ‐glutamyltranspeptidase, releasing SMC for subsequent uptake by other tissues (Li et al. 2025) (Figure 4).

**FIGURE 5:**
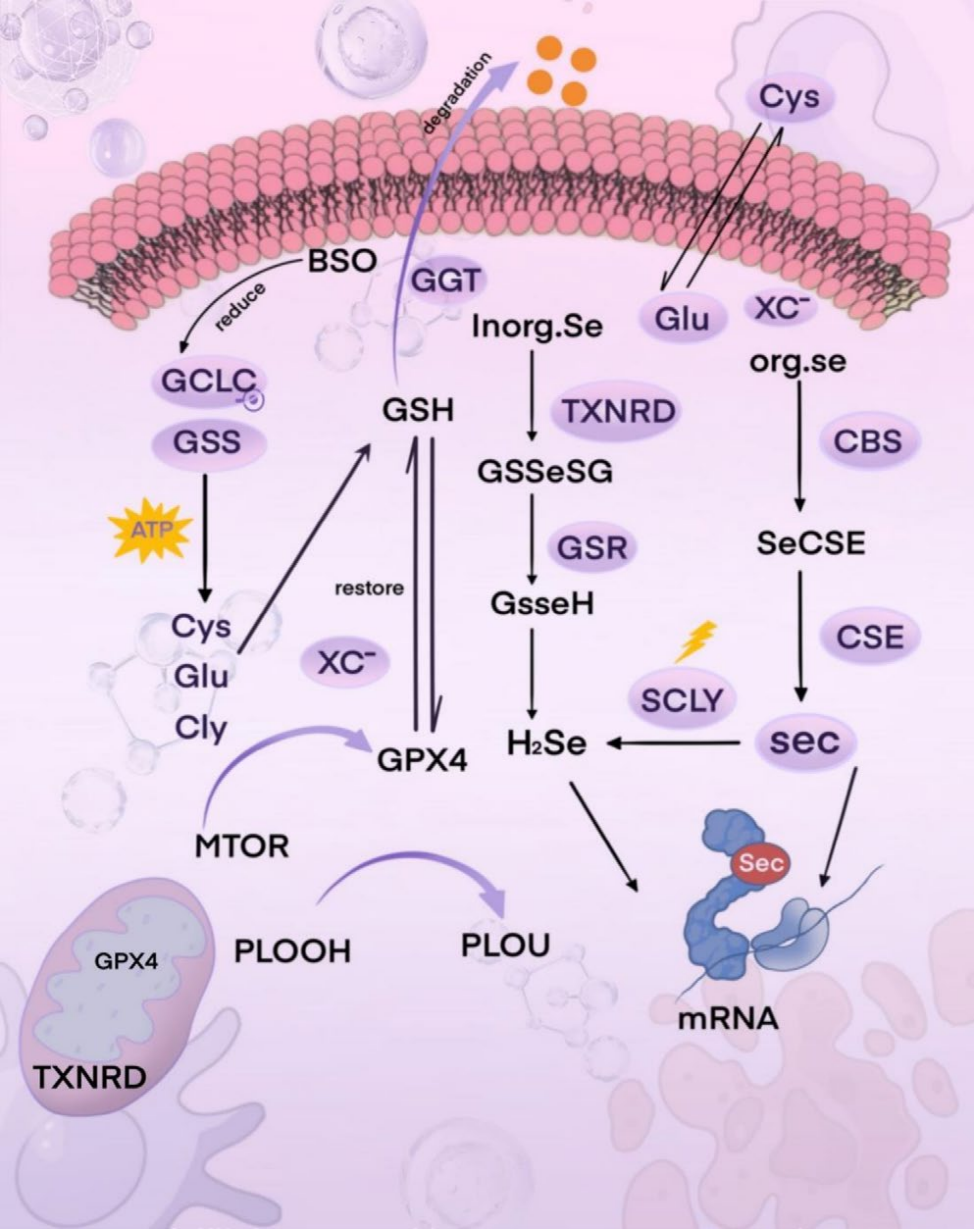
Cells maintain redox balance through the synergistic interplay of the glutathione system and selenium metabolic pathways, with GPX4 and transporters serving core functions.

In selenium metabolism, the enzymes cystathionine β‐synthase (CBS), cystathionine γ‐lyase (CGL), and selenocysteine lyase (SCLY) also participate in this trans‐selenation pathway, generating selenocysteine (Sec) from SeMet metabolism (Geillinger et al. 2014). Furthermore, selenocysteine or its derivatives (SMC/GSMC), derived from the degradation of selenoproteins (SELENOP) or proteins containing SeMet, can be recycled to regenerate HSe^−^. CBS and CGL typically generate sulfide (H_2_S^−^) in the transsulfuration pathway; intracellular selenium metabolism utilizes the same enzymes within this system (Wang, Chu, and Lin 2021). CBS can catalyze the conversion of SeMet to selenocystathionine (SeCSE), which is subsequently converted to Sec by CGL. Ultimately, Sec is converted by the SCLY enzyme into H_2_Se. This H_2_Se is then delivered to selenophosphate synthetase 2 (SEPHS2) and incorporated as the amino acid Sec during translation into selenoproteins via its dedicated tRNA (Sec tRNA[Ser]Sec) (Figure 5). Selenium excretion relies on methylation, whereby HSe^−^ is converted to MSe, which is subsequently transformed into DMSe and TMSe. These compounds are ultimately eliminated via exhalation through the lungs or renal excretion in urine. Excess selenomethionine (SeMet) is converted to MSe for excretion, primarily through the action of β − lyase or via S‐adenosylmethionine (SAM)‐dependent methylation (Ha et al. 2019; Song et al. 2018).

It is noteworthy that the synthesis of selenoproteins is a tightly regulated and prioritized biological process. When the selenium content in the body exceeds physiological requirements, the excess selenium typically undergoes methylation modification or conversion into metabolites such as selenosugars, followed primarily by excretion via the urinary system. However, the precise molecular mechanisms and detailed steps underlying this excretion process are not yet fully understood (Rayman et al. 2008; Evans et al. 2017). Intracellular selenium metabolism involves multiple critical steps and collaborates with the glutathione system to maintain redox homeostasis.

### 6.2 Selenium Metabolism in Solid Tumors

In the intricate microenvironment of solid tumors, the metabolic process of selenium diverges substantially from that in the normal physiological state. However, the exact mechanisms by which the invasiveness and malignant traits during tumorigenesis and tumor progression impact this complex selenium metabolism process are still under intensive investigation. The underlying mechanisms and detailed molecular pathways remain to be fully elucidated. A plethora of studies are focused on revealing the distinctive features of selenium metabolism in tumors and its potential therapeutic implications.

For instance, research into the mechanism of selenium uptake by tumor cells has shown that inorganic selenite can exert cytotoxic effects on cancer cells, and this process hinges on the x(c)‐cystine transporter (Olm et al. 2009). In an experiment, selenium nanoparticles (SeNPs) containing selenite were administered intraperitoneally to cancer cells implanted in the abdominal cavity of mice. The results demonstrated that the cancer cells were inactivated by the reactive oxygen species (ROS) generated by SeNPs, thereby offering a potential approach and direction for the application of selenium in cancer treatment (Wu et al. 2019).

Varlamova et al. investigated the effects of selenium nanoparticles (SeNPs) on four human cancer cell lines: A‐172 (glioblastoma), Caco‐2 (colorectal adenocarcinoma), DU‐145 (prostate cancer), and MCF‐7 (breast cancer). They discovered that SeNPs induced apoptosis in cancer cells in a concentration‐dependent manner, without triggering necrosis. Additionally, SeNPs enhanced the expression of pro‐apoptotic genes in nearly all cancer cell lines except Caco‐2 and activated diverse pathways within the adaptive and pro‐apoptotic signaling cascades of the unfolded protein response (UPR) (Varlamova et al. 2021). This finding may represent a significant strategy for the utilization of selenium nanoparticles in cancer treatment.

Selenium nanoparticles, along with methylselenic acid (MSA) that is prevalently found in organic selenium‐containing compounds, have demonstrated unique potential for cancer treatment. The underlying mechanism of action is principally rooted in selective cytotoxicity. MSA enhances the generation of reactive oxygen species (ROS) within cancer cells while depleting glutathione (GSH). This process leads to the production of methylselenol and circumvents the SCLY pathway, thereby impeding the survival and proliferation of cancer cells (Varlamova and Turovsky 2021).

Carlisle et al. have shown that selenium‐avid cancer cells employ SLC7A11 (a constituent of the cystine/glutamate antiporter SLC7A11) and xCT to facilitate increased selenium uptake (Carlisle et al. 2020), This, in turn, enables cancer cells to synthesize GPX4. GPX4 plays a crucial role in inhibiting ferroptosis, which represents a significant mechanism by which cancer cells resist cell death.

Within cancer cells, apart from the xCT transporter's role in promoting selenium metabolism, there are several enzymes that influence the selenium metabolism pathway, including SEPHS2, CSE, and CBS. These enzymes exert non‐trivial effects on the survival and reproduction of cancer cells. Notably, existing reports have indicated that CBS is upregulated in androgen‐dependent prostate cancer, colon cancer, and ovarian cancer cells (Bhattacharyya et al. 2013; Szabo et al. 2013), In HCC (hepatocellular carcinoma), prostate cancer, and glioma cell lines, the endogenous hydrogen sulfide (H_2_S) generated by CSE participates in the survival and proliferation of these malignant cell lines (Youness et al. 2021).

#### 6.2.1 Role of Selenium Phosphate Synthase 2 (SEPHS2) in Selenium Metabolism and Cancer

Selenophosphate synthetase 2 (SEPHS2), a pivotal enzyme within the selenocysteine biosynthesis pathway, is indispensable for the survival of cancer cells. An increase in the intracellular uptake of selenocysteine (Sec) results in elevated production of selenide. As a toxic intermediate metabolite to cancer cells, the accumulation of selenide jeopardizes their survival. To effectively counteract the toxicity of H_2_Se, cancer cells depend on the selenoprotein SEPHS2 for detoxification, a process that is essential for maintaining their viability.

Experimental evidence has demonstrated that when breast cancer cells (MDAMB231) with the SEPHS2 gene knocked out are inoculated into wild‐type nude thymic mice, the growth of in situ breast tumor xenografts is markedly inhibited (Carlisle et al. 2020). This finding clearly elucidates the critical role of SEPHS2 in the mechanism promoting selenium metabolism in cancer cells. Moreover, it presents a highly promising new target and direction for the innovation of cancer treatment strategies, thereby laying a theoretical groundwork for the subsequent development of anti‐cancer drugs or therapeutic approaches targeting SEPHS2.

#### 6.2.2 Role of Cystathionine Gamma Enzyme (CSE) in Selenium Metabolism and Cancer

Cystathionine‐γ‐lyase (CSE), an enzyme responsible for catalyzing the production of hydrogen sulfide (H_2_S), exhibits abnormal expression patterns that are intricately linked to tumorigenesis, tumor progression, and angiogenesis. A wealth of research has demonstrated that the endogenous H_2_S generated by CSE has the capacity to drive the proliferation of human cancer cells. Notably, the STAT3 and VEGF signaling pathways play critical roles in this promotional mechanism (Deng et al. 2022; Wang, Shi, Liu, et al. 2019). Specifically, elevated CSE expression levels have been firmly established to be closely associated with breast cancer progression related to the STAT3 signaling pathway. CSE can facilitate the malignant biological behaviors of cancer cells, such as proliferation, survival, and migration, by activating a cascade of downstream signaling molecules (You et al. 2017).

In recent years, with the increase of research into the pathogenesis of cancer, the inhibition of CSE and the endogenous H_2_S it generates has emerged as a novel focus in the realm of cancer treatment research. For instance, a novel cystathionine‐γ‐lyase inhibitor, I194496, has been identified to effectively impede the growth of human triple‐negative breast cancer cells by concurrently suppressing the PI3K/Akt (phosphatidylinositol 3‐kinase/protein kinase B) and Ras/ERK (rat sarcoma viral oncogene/extracellular regulated protein kinases) signaling pathways. Additionally, it can markedly reduce the metastatic potential of cancer cells by inhibiting the STAT3 and VEGF signaling pathways (Liu et al. 2021).

Another inhibitor, I157172, decreases the growth, proliferation, and migration rates of MCF7 breast cancer cells in a dose‐dependent manner via sirt1‐mediated STAT3 deacetylation (Wang, Shi, Zhang, et al. 2019). These research findings comprehensively indicate that further exploration of the mechanism through which CSE participates in H_2_S metabolism within cancer cells and its influence on the biological behaviors of cancer cells holds significant theoretical importance and potential clinical application for the development of innovative and effective cancer treatment modalities.

#### 6.2.3 Role of Cystathionine Beta‐Synthase (CBS) in Selenium Metabolism and Cancer

Cystathionine β‐synthase (CBS) is an enzyme of paramount importance in cellular metabolism, having critical regulatory functions. It not only partakes in the metabolic regulation of homocysteine (Hcy) but also assumes a pivotal role in the biosynthesis of hydrogen sulfide (H_2_S). Existing research has revealed that CBS‐driven endogenous H_2_S production promotes tumor growth via multiple intricate molecular mechanisms. These mechanisms include, but are not restricted to: (i) maintaining the stability of mitochondrial respiration and ATP synthesis, thereby furnishing an ample energy supply for the rapid proliferation of cancer cells; (ii) directly stimulating the proliferation and survival signaling pathways in cancer cells, enhancing their anti‐apoptotic capabilities; (iii) participating in the regulation of intracellular redox balance, thereby creating a microenvironment conducive to cancer cell growth; (iv) facilitating vasodilation, which improves the blood supply to tumor tissues, thus providing the essential nutritional support and material exchange conditions for tumor growth and metastasis (Zhu et al. 2018).

Data from multiple pre‐clinical studies show that compared with adjacent normal tissues or non‐transformed cells, the expression levels of CBS in various solid tumor tissues or cell lines such as colon cancer (Phillips et al. 2017), ovarian cancer (Bhattacharyya et al. 2013), prostate cancer (Guo et al. 2012) and breast cancer (Sen et al. 2015), showed a trend of significantly higher expression levels compared with neighboring normal tissues or non‐transformed cells. This phenomenon further confirms the important role of CBS in the formation and development of solid tumors. However, despite the current recognition of the importance of CBS in tumorigenesis and development, research on the relationship and specific mechanism of action between selenium and CBS remains relatively scarce.

In a study exploring the influence of selenium deficiency on the liver metabolome of male mice, it was observed that downregulation of CBS expression led to pronounced perturbations in lipid and one‐carbon metabolism within the mouse liver. Additionally, when the selenium intake in the animal diet was decreased to half of the recommended level, the activities of total glutathione peroxidase (GPX) and thioredoxin reductase (TXNRD) in the liver declined accordingly (Geillinger et al. 2014). These metabolic derangements and alterations in enzyme activities potentially augment the risk of cancer development. Nevertheless, the precise molecular mechanisms and the role played by selenium in this context remain incompletely elucidated. Thus, further in‐depth and systematic investigations are warranted to clarify their underlying relationships and functional principles.

Santos et al. carried out a study on the effects of SeChry and folic‐acid‐targeted fourth‐generation polyurea dendrimer (SeChry@PUREG4‐FA) nanoparticles on three distinct ovarian cancer cell lines (ES2, OVCAR 3, and OVCAR 8) and two non‐malignant cell lines (HaCaT and HK 2). The findings demonstrated that SeChry exerted specific inhibitory effects on CBS in ovarian cancer and exhibited relatively low toxicity compared to other cell types (Santos, Ramos, et al. 2019). However, this study has yet to assess the specific impact of SeChry on the production of H_2_Se.

## 7 Autophagy‐Regulated GPX4 Homeostasis Coordinates Iron Prolapse and Cancer

In the realm of solid tumor research, a wealth of findings has elucidated that GPX4 in cancer cells assumes a central role in countering ferroptosis‐induced cell death, thereby serving as a critical determinant for the survival of cancer cells (Dixon et al. 2012). Ferroptosis, an iron‐dependent, non‐apoptotic mode of cell death, was initially introduced in 2012. It diverges from other programmed cell death modalities, including necrotic death, apoptosis, and autophagy. Currently, it is well recognized that the canonical ferroptosis‐inhibitory pathway principally comprises the Xc^−^/GSH/GPX4 axis. This axis represents a surveillance mechanism that impedes ferroptosis and promotes tumorigenesis by converting potentially toxic phospholipid hydroperoxides into non‐toxic lipid alcohols (Weaver and Skouta 2022).

Specifically, system Xc^−^, a heteromeric antiporter, transports cystine into cells. Once inside, the cystine participates in the biosynthesis of glutathione (GSH). The synthesized GSH then functions as an essential cofactor for GPX4, collaborating in this process (Seibt et al. 2019). As a pivotal regulator of ferroptosis, GPX4 is capable of directly suppressing the accumulation of lipid peroxides within cells. By doing so, it effectively prevents the generation of reactive oxygen species (ROS) and maintains intracellular redox equilibrium, which is indispensable for cancer cells to elude the fate of ferroptosis (Yang, SriRamaratnam, et al. 2014).

Given that GPX4 is a selenoprotein and its synthesis is intricately dependent on cellular selenium uptake, ferroptosis is widely acknowledged as a selenium‐dependent cell‐death mechanism (Friedmann Angeli and Conrad 2018). Recent research into the mechanisms by which cancer cells resist ferroptosis has shown that SELENOP, acting as a regulatable selenium source within cancer cells, can be internalized by cells via the receptor LRP8 and subsequently degraded within lysosomes. This process exerts a profound influence on selenium homeostasis within cancer cells, as well as on the synthesis and functionality of selenoproteins. For example, the research by Li et al. has demonstrated that in a low‐selenium environment, the translation of GPX4 is markedly disrupted. Ribosome stalling and premature translation termination occur, thereby impeding the synthesis of the GPX4 protein (Li et al. 2022). This finding implies that the selenoprotein hierarchy in cancer cells has been altered, giving rise to cancer‐specific vulnerabilities. Consequently, cancer cells can be rendered sensitive to ferroptosis through targeted agents or autophagy‐mediated GPX4 degradation.

Specifically, enhanced ferroptosis resulting from GPX4 inhibition can augment the sensitivity of colorectal cancer to oxaliplatin. In non‐small‐cell lung cancer, it can heighten the sensitivity of cancer cells to lapatinib. In hepatocellular carcinoma, it can improve the sensitivity of cancer cells to sorafenib. In Epstein–Barr virus‐infected nasopharyngeal carcinoma, it can increase the sensitivity of cancer cells to platinum‐based drugs (Yang et al. 2021; Wang, Bin, et al. 2021; Yuan et al. 2022; Ni et al. 2021).

Macroautophagy (hereafter simply referred to as “autophagy”) is a membrane‐mediated biological process. In a highly context‐dependent manner, autophagy can confer cellular protection against diverse pathogens. However, it can also promote cell‐death induction by releasing lysosomal hydrolases or selectively dismantling anti‐cell‐death proteins (Doherty and Baehrecke 2018). Autophagy mediated by Cu^2+^ and erastin plays a pivotal role in GPX4 degradation. This strategy of resensitizing cancer cells to ferroptosis has been demonstrated to be highly efficacious in numerous experiments.

**FIGURE 6:**
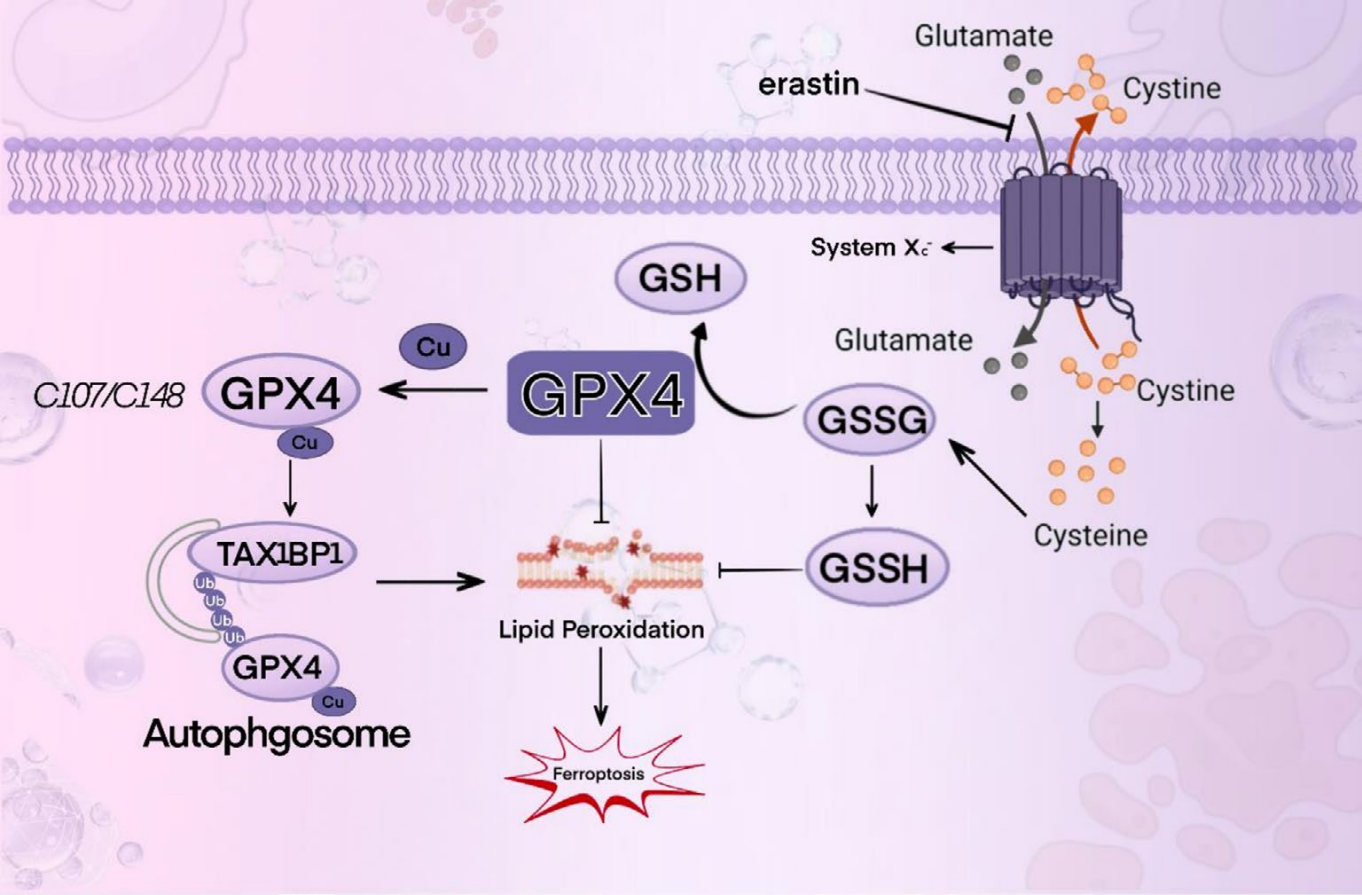
Copper‐mediated autophagic degradation of GPX4 amplifies ferroptosis.

**FIGURE 7:**
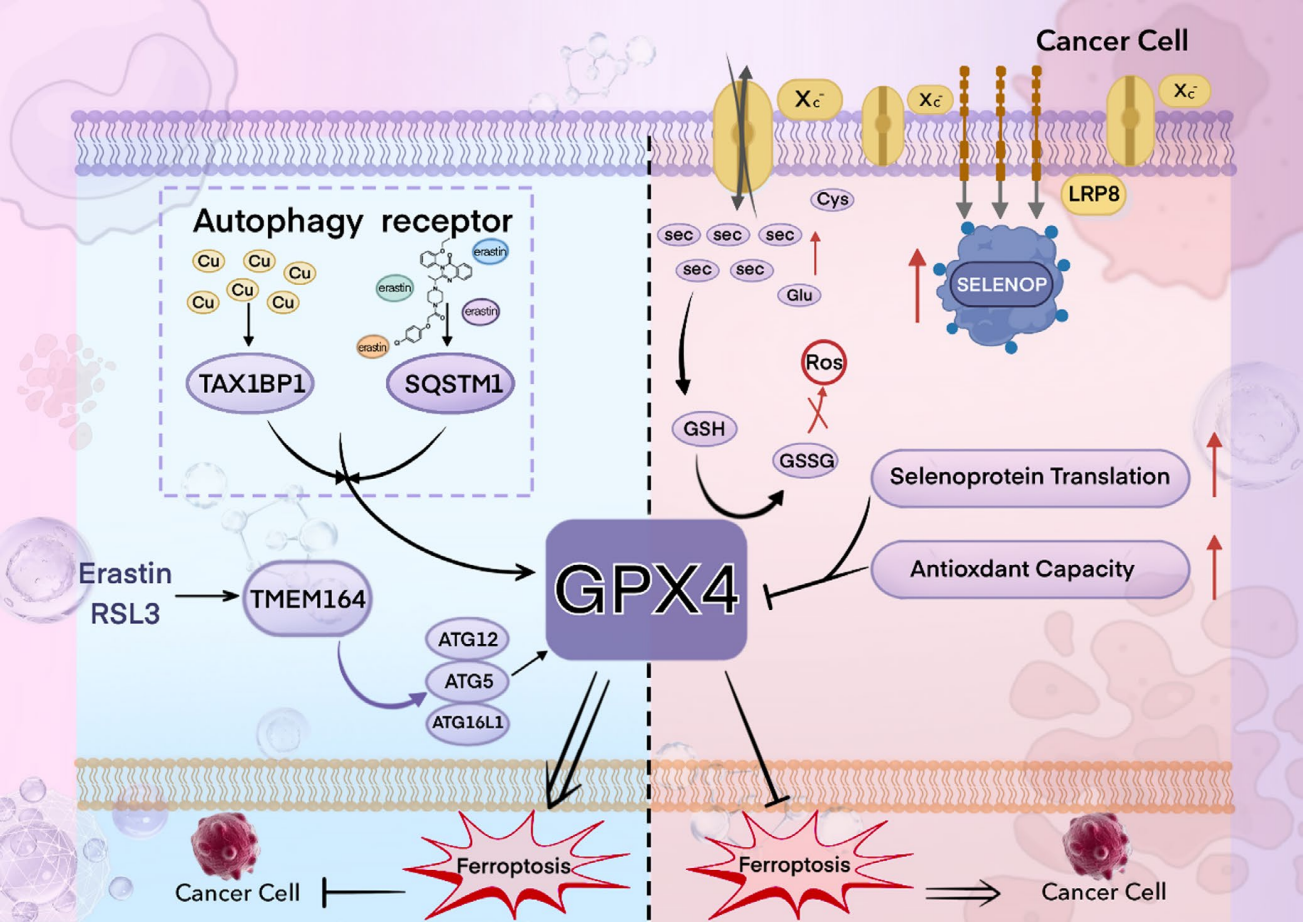
Mechanisms of GPX4 regulation in cancer cell ferroptosis. Left panel: Copper and erastin promote GPX4 degradation via TAX1BP1 and SQSTM1, respectively. In specific cancer cells, TMEM164 facilitates the formation of relevant complexes, promoting GPX4 degradation induced by erastin or RSL3. This process enhances ferroptosis and suppresses cancer cell growth. Right panel: Cancer cells can increase selenium uptake to promote selenoprotein translation and elevate.

Erastin induces ferroptosis by specifically inhibiting the cystine/glutamate antiporter System Xc^−^ (composed of SLC7A11 and SLC3A2 subunits), thereby blocking extracellular cystine uptake. This leads to intracellular cysteine depletion and disruption of glutathione (GSH) biosynthesis. The loss of GSH results in functional impairment of the key antioxidant enzyme GPX4 (glutathione peroxidase 4), which depends on GSH's reducing activity. Consequently, phospholipid hydroperoxides (PL‐OOH) accumulate abnormally. In the presence of free ferrous ions (Fe^2+^), the Fenton reaction catalyzes massive generation of reactive oxygen species (ROS), driving lipid peroxidation chain reactions in polyunsaturated fatty acids (PUFAs). This ultimately disrupts cellular membrane integrity and triggers ferroptosis (Xue et al. 2023; Dos Santos et al. 2023). Notably, this cell death pathway can be completely suppressed by ferroptosis‐specific inhibitors (e.g., Ferrostatin‐1), but remains unresponsive to inhibitors of apoptosis or necroptosis, confirming the distinct molecular nature of this mechanism (Xue et al. 2023) (Figure 6). Concurrently, Xue Qian et al. investigated the mechanism of Cu^2+^‐mediated ferroptosis and its effect on GPX4 protein expression. Their study revealed that Cu^2+^ increases susceptibility to ferroptosis by inducing TAX1BP1‐mediated autophagic degradation of GPX4, a process that is ROS‐independent and targets TAX1BP1 as the key autophagy receptor for GPX4 degradation (Xue et al. 2023). In contrast to TAX1BP1, SQSTM1 mediates GPX4 degradation under conditions induced by erastin. Mechanistically, exogenous Cu^2+^ directly binds to cysteine residues (specifically Cys107 and Cys148) within GPX4, promoting its ubiquitination and aggregation formation. This modification subsequently facilitates recognition by TAX1BP1 (Chen et al. 2023). Furthermore, the transmembrane protein TMEM164 has emerged as a novel key determinant of autophagy‐dependent ferroptosis. Similar to TAX1BP1 and SQSTM1, TMEM164 plays a significant role in regulating GPX4 degradation. TMEM164 localizes to the plasma membrane and various cellular organelles. In certain cancer cells, the formation of the ATG12‐ATG5‐ATG16L1 complex and the subsequent degradation of GPX4 induced by ferroptosis activators (such as erastin or RSL3) requires the involvement of TMEM164 (Liu, Liu, et al. 2023). This groundbreaking research elucidates the bridging role of the transmembrane protein TMEM164 between autophagy and ferroptosis. It establishes, for the first time, a previously underrecognized molecular link between these two processes (Figure 7).

The strategy in which Cu^2+^ and erastin induce autophagy‐mediated degradation of GPX4—thereby reversing cancer cell resistance to ferroptosis—reveals a novel anticancer modality. This mechanism further suggests that, beyond developing small‐molecule agents that directly target GPX4, the pathway can be modulated indirectly through precision nutritional interventions. For example, when formulating foods for special medical purposes (FSMPs) for oncology patients, one might leverage specific selenium species or tune their bioavailability to gently adjust the activity threshold of GPX4, creating an intracellular milieu more favorable to conventional therapies.

From a translational and regulatory perspective, China's National Food Safety Standard GB 1903.12‐2015 authorizes Se‐methyl‐L‐selenocysteine (SeMSC) as a nutrient fortifier; in the United States, under 21 CFR Part 573, the FDA has progressively expanded permitted selenium sources for feeds (e.g., zinc‐L‐selenomethionine and hydroxy analogs of selenomethionine) and, in 2025, added zinc‐L‐selenomethionine for broiler feeds; within the European Union, Directive 2002/46/EC regulates the addition of vitamins and minerals to foods, and EFSA has established tolerable upper intake levels for selenium. In Canada, the Natural Health Products Ingredients Database (NHPID) lists selenium—in the form of selenocysteine—as an acceptable medicinal ingredient and includes selenium yeast as an entry for NHP formulations. Collectively, these frameworks facilitate the safe, effective, and compliant market introduction of innovative selenium‐ and selenocysteine‐based foods, including FSMPs.

## 8 Conclusion

This review examines selenium metabolism and its key effector—glutathione peroxidase 4 (GPX4)—with a focus on its central role in autophagy‐regulated ferroptosis in solid tumors. These advances not only enrich fundamental understanding but also inform innovation in the food industry—particularly in foods for special medical purposes (FSMPs) and functional foods and lay the groundwork for novel anticancer strategies.

Given the widespread overexpression of GPX4 across multiple cancer types, leveraging Cu^2+^ and erastin to induce autophagy‐dependent GPX4 degradation and thereby sensitize cancer cells to ferroptosis has emerged as a promising therapeutic paradigm that may surpass conventional approaches. The selenium metabolism–GPX4–ferroptosis axis delineated herein provides actionable targets for drug discovery and a theoretical basis for precision nutrition design in oncology‐oriented FSMPs. Accordingly, product developers and clinical dietitians should comply with China's food‐safety standards for L‐Se‐methylselenocysteine and consult international frameworks, such as the U.S. FDA's regulation of nutrient fortification and the EU EFSA's tolerable upper intake levels for selenium—to ensure scientific rigor, safety, and efficacy.

It should be noted that the interplay between GPX4 and ferroptosis involves complex signaling pathways and molecular interactions, and current understanding remains preliminary. Future work integrating in‐depth basic research with well‐designed clinical trials is warranted to substantiate its translational and clinical value.

## Author Contributions

**Liang Yang:** writing – review and editing (equal). **Yu Jiang:** writing – original draft (equal), writing – review and editing (equal). **Haoyue Wu:** writing – original draft (equal), writing – review and editing (equal). **Jinnan Sun:** visualization (equal). **Xinyue Huang:** visualization (equal). **Jingfeng Hong:** visualization (equal). **Bin Zheng:** writing – review and editing (equal). **Jinmiao Zhu:** writing – review and editing (equal).

## Conflicts of Interest

The authors declare no conflicts of interest.

## Data Availability

The authors have nothing to report.
